# FDX1 as a predictive biomarker and therapeutic target for lymph node metastasis in gastric cancer

**DOI:** 10.1007/s10238-026-02160-0

**Published:** 2026-05-10

**Authors:** Tianhao Zhang, Jianqiu Liu, Peng Duan, Zikang Li, Zhixin Huang, Zeyu Zhao, Yang Cheng, Zhi Liang, Jianhui Chen, Meilan Cai, Jinping Ma

**Affiliations:** 1 Center of Gastrointestinal Surgery, the First Affiliated Hospital, Sun Yat-Sen University, Guangzhou, China; 2 Gastric Cancer Center, Sun Yat-Sen University, Guangzhou, China; 3 Laboratory of General Surgery, the First Affiliated Hospital, Sun Yat-Sen University, Guangzhou, China; 4 Professional Foreign Language Teaching and Research Office, Inner Mongolia Medical University Affiliated Hospital, Hohhot, China

**Keywords:** Gastric cancer, Cuproptosis-related genes, Lymph node metastasis, Tumor immune microenvironment, Molecular subtypes

## Abstract

The prognostic values of cuproptosis-related genes (CRGs) in gastric cancer with lymph node metastasis (GCLM), especially in the tumor immune microenvironment (TIME), remain unclear. We analyzed the expression, mutation, immunity, drug sensitivity, and prognostic value of CRGs in GCLM using TCGA and GEO cohorts. Consensus clustering was performed to identify CRG subtypes, with differences characterized by multi-omics analysis. A CRG-based prognostic risk score and immune score were constructed for individualized assessment, and the role of CRGs was validated through in vitro and in vivo experiments. Consensus clustering revealed that CRGs were significantly enriched in biological processes related to mitosis and energy metabolism, as well as in immune-related and cancer-associated pathways. Four distinct CRG subtypes were identified, showing marked differences in expression profiles, prognosis, genetic alterations, TIME, and chemotherapeutic drug sensitivity. We developed an exploratory CRG-based prognostic risk score for preliminary individualized assessment, and the functional relevance of CRGs in GCLM was further validated through in vitro experiments. Among these, FDX1, LIAS, DLAT, MTF1, and GLS were identified as key determinants of overall survival in patients with GCLM, with FDX1 emerging as a potential independent prognostic factor. Notably, upregulation of FDX1 significantly suppressed lymph node metastasis of gastric cancer cells in a mouse popliteal lymph node metastasis model. Our data uncovers FDX1 might be a potential favorable prognostic factors in GCLM patients. These findings may improve our understanding of CRGs in GCLM and provide new in-sights for assessing prognosis and developing more effective treatment strategies.

**Supplementary Information:**

The online version contains supplementary material available at 10.1007/s10238-026-02160-0.

## Plain language summary

Lymph node metastasis represents the most frequent metastatic pattern in gastric cancer; however, the specific molecular mechanisms underlying this process remain largely elusive. Although the roles for cuproptosis related genes (CRGs) in tumorigenesis and progression have been confirmed, their clinical application value in gastric cancer with lymph node metastasis (GCLM) remain unclear. This study formulated the expression, genetic alteration, immune cell infiltration and prognostic values of CRGs in GCLM and provides a new direction for the individualized diagnosis and treatment of GC. A new and effective prognostic model of CRGs in patients with GCLM was established. We believe that our study makes a significant contribution to the literature because distinct correlations were found among the expression levels of CRGs and the infiltration of different immune cells. These results indicate that CRGs might significantly be correlated with oncogenesis and the progression of GC. In addition, FDX1 might be a favorable prognostic factor for patients with GC through in vitro and in vivo experiments.

**Supplementary Information:**

The online version contains supplementary material available at 10.1007/s10238-026-02160-0.

## Introduction

Gastric cancer (GC) emerged as a significant cause of mortality, ranking fourth among cancer-related deaths globally. Furthermore, it established itself as the fifth most prevalent malignant neoplasm worldwide [1]. Lymph node metastasis (LM) is the most common pattern of metastasis in GC and is strongly associated with poor prognosis. Currently, GC accounts for over one million new cases annually worldwide, with China contributing approximately half of this global burden. In China, a substantial proportion of patients, up to 90%, are initially diagnosed at advanced stages. In 2022 alone, China reported 358,700 new cases of gastric cancer and 260,400 related deaths [2, 3]. This statistic highlights the concerning situation where patients are already in advanced stages of the disease when they receive their diagnosis. Moreover, the molecular characteristics of GC Lymph node metastasis (GCLM) remain unknown, and there is no definitive consensus on the necessity of lymph node dissection for early GC. Therefore, improving the survival of GCLM patients requires the identification of effective therapeutic targets.

Cuproptosis (means “copper death”) is a newly identified, mitochondria-dependent form of programmed cell death, distinct from other oxidative stress-related modes such as apoptosis, ferroptosis, and necroptosis. Research has revealed that cuproptosis is critically involved in tumorigenesis, progression, and metastasis, underscoring its potential as a therapeutic target [4, 5].All organisms require copper as a cofactor, but excessive accumulation of free copper can lead to cell death [6]. Although the mechanisms of the toxicity of common metals are well established, how excess copper induces cell death and the mechanisms underlying copper-induced cytotoxicity remain unclear [7–9]. Copper ionophore-induced cell death has been demonstrated across hundreds of cell lines to involve intracellular copper accumulation [7, 10]. Under conditions of copper excess, lipoylated proteins may aggregate and become destabilized, leading to protein toxicity stress and ultimately cell death [6]. Numerous reports have indicated that tumors require higher levels of copper than healthy tissues, further highlighting the role of copper in cancer development [11]. The precise mechanisms by which cuproptosis contributes to lymph node metastasis in GC have yet to be elucidated, despite its recognized role in tumorigenesis and progression.

A systematic study of cuproptosis-related genes (CRGs) will contribute toward exploring the molecular mechanisms associated with copper and the malignant advancement of GC. CRGs mainly comprise 10 key genes—FDX1, DLD, LIAS, DLAT, LIPT1, PDHA1, PDHB, MTF1, GLS, and CDKN2A, which were derived from a broader set of 173 cuproptosis-related genes. This expanded gene set was originally identified based on the intersection of two structurally distinct copper-loaded ionophores (elesclomol and the active form of disulfiram, diethyldithiocarbamate) and filtered using a significance threshold of FDR < 0.01 [6]. Research indicates that FDX1 and protein lipoacylation are involved in copper ion carrier-induced death of cells [6]. Recently, elevated copper concentrations have been reported in patients with various cancers, including oral [12], thyroid [13], breast [14, 15], lung [16, 17], gastrointestinal [18, 19], prostate [20], gall bladder [21], and gynecologic cancers [22]. Copper imbalance affects changes in mitochondrial respiration, glycolysis, insulin resistance, and lipid metabolism [23–25]. In addition, copper can regulate autophagy through ULK1 and ULK2 [26].

In this study, we integrated publicly available datasets from the Gene Expression Omnibus (GEO) and The Cancer Genome Atlas (TCGA) with in vitro and in vivo experiments to comprehensively investigate the associations between CRGs and GCLM. Our analyses encompassed expression profiles, prognosis, genomic alterations, immune infiltration, immune scores, chemotherapeutic drug sensitivity, and clinicopathological features. This study aims to establish a preliminary predictive model for lymph node metastasis in early gastric cancer, with the aim of supporting personalized clinical decision-making and reducing the need for unnecessary extended lymphadenectomy.

## Materials and methods

### Patients and datasets

The dataset and clinical data for stomach adenocarcinomas (STAD) comprising 375 cases were acquired from the TCGA database, along with an additional 36 paracancer samples. The GEO database contains RNA-seq data and survival information for 875 STAD samples (GSE15459, GSE14210, GSE22377, GSE29272, GSE51105, and GSE62254). Detailed description of preprocessing steps for each dataset: 1) TCGA-STAD dataset: Raw HTSeq-FPKM counts were obtained from the GDC Data Portal. Genes with low expression (average FPKM < 1 across all samples) were filtered out. The data were then log₂-transformed and quantile-normalized to ensure comparability across samples.Only patients with pathologically confirmed lymph node metastasis (pN1, pN2, or pN3) were included as GCLM cases (246 patients); 2) GEO datasets (GSE15459, GSE14210, GSE22377, GSE29272, GSE51105, and GSE62254): These datasets were obtained from the GEO database and downloaded in MINiML format, which contains the platform, sample, and complete GSE record information for each dataset. For datasets that had not been normalized, we applied log2 transformation. When standardization was required, we used the “normalize.quantiles” function from the “preprocessCore” package in R to perform quantile normalization. Probe IDs were converted into gene symbols based on the corresponding platform annotation files; probes mapping to multiple genes were excluded, and for genes with multiple probes, the average expression value was calculated. To address batch effects within the same dataset and platform, we applied the “removeBatchEffect” function from the “limma” package. For integrated analyses involving multiple datasets or different platforms within the same dataset, we first extracted the common gene symbols across datasets, designated each dataset or platform as a separate batch, and again used “removeBatchEffect” to eliminate batch-related variation. The effectiveness of data normalization was assessed using box plots, while batch effect removal was evaluated by comparing PCA plots before and after batch correction. Demonstration of effective batch effect removal: The effectiveness of data normalization was assessed using box plots, while batch effect removal was evaluated by comparing principal component analysis (PCA) plots before and after batch correction. Published literature was used to compile lists of CRGs [6]. We also collected cancerous and adjacent tissues from 59 gastric cancer patients at the First Affiliated Hospital of Sun Yat-sen University to validate our findings. The TCGA-STAD GCLM subset served as the training and internal test cohort. All GEO datasets were used exclusively for external validation; no model tuning or feature selection was performed on these datasets. The institutional cohort was used solely to validate FDX1 expression differences and was not used for prognostic model evaluation. This study was approved by the Institutional Review Board of the First Affiliated Hospital of Sun Yat-sen University and conducted according to the tenets of the Declaration of Helsinki. Each participant signed a consent form before participating in the study.

### Prognostic model development and validation strategy

In this study, the GEPIA 2.0 [27], UALCAN [28], and The Human Protein Atlas [29] databases were used to explore gene expression profiles and prognostic factors in CRGs. We used R version 4.0.3 (R Foundation for Statistical Computing, Vienna, Austria) and the Kaplan–Meier Plotter [30] database for prognostic analysis. We downloaded STAR-counts data and corresponding clinical information for Gastric cancer from the TCGA database (https://portal.gdc.cancer.gov). The data were converted to transcripts per million (TPM) and normalized using log2 (TPM + 1) transformation. After retaining samples with both RNA‑seq and clinical data, a total of 246 samples were selected for further analysis. Feature selection was performed using the least absolute shrinkage and selection operator (LASSO) regression algorithm with tenfold cross‑validation, implemented via the glmnet package in R. A multivariate Cox regression analysis was then conducted using the survival package to construct the prognostic model. An iterative stepwise selection procedure was applied to identify the optimal model. Kaplan‑Meier curves were generated, with p‑values and hazard ratios (HR) along with 95% confidence intervals (CI) derived from log‑rank tests and univariate Cox proportional hazards regression. All statistical analyses were performed using R software version 4.0.3, and a p‑value < 0.05 was considered statistically significant. The CRGs gene signature was then applied without any further tuning to six independent GEO datasets (https://kmplot.com/analysis/) (GSE15459, GSE14210, GSE22377, GSE29272, GSE51105, GSE62254). For each dataset, we calculated the risk score using the identical formula, stratified patients into high and low risk groups using the same cutoff (median risk score from the training cohort), and performed survival analysis. We also validated FDX1 expression in our own independent cohort of 59 paired gastric cancer tissues.

### Unsupervised clustering of CRGs to identify differentially expressed genes (DEGs)

The ‘ConsensusClusterPlus’ R package (v1.54.0) was used for consensus clustering analysis and identifying CRG expression clusters. It was possible to draw 100 samples from the total sample, and the maximum number of clusters was 6. For generating cluster heatmaps, we used the R package ‘pheatmap’ (v1.0.12). The DEGs between the different subtypes were identified with the R package limma [31]. To indicate the potential functions of the DEGs and enriched pathways, we used the ‘clusterProfiler’ [32] package for functional enrichment analysis.

### Genomic alteration and drug susceptibility analysis

We identified the genomic alteration profiles of CRGs using the cBioPortal database [33, 34]. The ‘maftools’ package in R was employed to ascertain the somatic mutation burden (TMB) scores, specifically for different subtypes of GC. Moreover, to investigate the therapeutic efficacy of chemotherapy drugs on distinct subtypes, the ‘pRRophetic’ package was utilized to determine semi-inhibitory concentrations (IC50).

### The construction and validation of a risk model based on CRGs

#### LASSO Cox regression analysis

A Least absolute shrinkage and selection operator (LASSO) Cox regression analysis was performed to construct a prognostic model using selected candidate cancer-related genes (CRGs). The prognostic model was built in R using the ‘glmnet’ package, which allowed for the identification of the most influential CRGs for predicting outcomes. A total of 10 candidate CRGs were included in the model selection process. These CRGs were carefully chosen based on their potential relevance to cancer prognosis [35]. In order to calculate the risk score formula, we used the following formula risk score = Σcoef × gene expression. Risk factors were compared using receiver operating characteristic (ROC) curves. Log-rank tests were used to compare survival differences between the different risk groups using Kaplan–Meier survival analysis. Several independent prognostic risk factors were identified using univariate and multivariate Cox regression analyses. To assess the prognostic value of the nomogram, a calibration plot was calculated using risk-related genes and clinicopathological features [36]. The selected dataset includes risk score, survival time, and survival status. The top panel shows a scatter plot of risk scores arranged from low to high, with different colors representing different risk groups. The middle panel displays the distribution of risk scores against survival time and survival status for each sample. The bottom panel presents a heatmap of gene expression for the features included in the signature. In the Kaplan‑Meier survival curves of this risk model applied to the dataset, comparisons between groups are performed using the log‑rank test. The hazard ratio (HR) for the high‑risk group indicates the risk of the high‑risk group relative to the low‑risk group. An HR > 1 suggests a risk model, whereas an HR < 1 suggests a protective model. The 95% confidence interval (CI) represents the confidence interval of the HR. Median time indicates the time at which the survival rate reaches 50% in each group (i.e., median survival time), expressed in years. Additionally, the time‑dependent ROC curves and their AUC values at different time points show that higher AUC values indicate stronger predictive ability of the model.

#### Nomogram analysis

First, univariate and multivariate Cox proportional hazards regression analyses were performed. Forest plots were generated using the forestplot package to visualize the P‑values, hazard ratios (HRs), and 95% confidence intervals (CIs) for each variable. Subsequently, based on the results of the multivariate Cox proportional hazards model, a nomogram was constructed using the rms package to predict the X‑year overall survival rate. All statistical analyses were conducted using R software (version 4.0.3). A P‑value < 0.05 was considered statistically significant. Variables that were significantly associated with prognosis in both univariate and multivariate analyses were identified as independent prognostic factors independent of other clinical variables. The predictive performance of the nomogram was evaluated using calibration curves; the closer the calibration curve to the diagonal line (ideal curve), the better the predictive accuracy of the model [37].

### qRT-PCR assay

The levels of cancer-related genes (CRGs) in gastric cancer (GC) tissue were determined through quantitative reverse transcription polymerase chain reaction (qRT-PCR) assay. To extract total RNA from GC tissues, the RNAiso kit (Takara, Kusatsu, Japan) was employed following the instructions provided by the manufacturer. The primers we use are as follows: FDX1 (Forward): TTCAACCTGTCACCTCATCTTTG; FDX1 (Reverse): TGCCAGATCGAGCATGTCATT.

### Immune cell infiltration analysis

To achieve reliable immune score assessment, we employed the immunedeconv R package and and TIMER [38] database, which integrates six state‑of‑the‑art algorithms for immune cell infiltration estimation: TIMER, xCell, MCP‑counter, CIBERSORT, EPIC, and quanTIseq. These algorithms have undergone systematic benchmarking, and each exhibits unique performance characteristics and advantages. All statistical analyses were performed using R software (version 4.0.3). A P‑value < 0.05 was considered statistically significant.

### Immunohistochemistry

Immunohistochemical analysis was conducted with the utilization of epitope retrieval method using 50 × TRIS–EDTA (pH 9.0). To minimize nonspecific binding, subsequent blocking step was performed using phosphate-buffered saline containing 20% goat serum and 0.25% TritonX-100. The samples were incubated with primary antibodies targeting FDX1 (12,592–1-AP; ProteinTech, Wuhan, China) for a duration of 16–18 h at 4 °C. Following this extended incubation, the samples underwent an additional incubation step with secondary antibodies for 60 min at room temperature in order to enable labeling.

### Cell culture

All gastric cancer cell lines (MKN1 and MGC803) were obtained from the Cell Bank of the Chinese Academy of Sciences (Shanghai, China). Cell line authentication was performed by the supplier using short tandem repeat (STR) profiling, and the most recent authentication certificates are available upon request. In our laboratory, cells were passaged for fewer than 3 months after resuscitation, and no further authentication was performed during this period. All cell lines were routinely tested for mycoplasma contamination using the Myco-Zero™ Mycoplasma Elimination Reagent (Beyotime, China) every 4 weeks and before each major experiment. Only mycoplasma-negative cells were used for experiments.

### Cell migration and invasion assays

In this study, we used the established human gastric cancer cell lines MKN1 and MGC803 to assess their metastatic capacity via Transwell migration and invasion assays. Cells were seeded at a density of 5 × 10^4^ cells per well in the upper chamber of 24-well Transwell plates (8-μm pore size, Corning, USA), suspended in 200 μL of serum-free medium with or without Matrigel coating. For invasion assays, the upper chamber was pre-coated with 30 μL of Matrigel (BD Biosciences, USA) diluted 1:9 in serum-free medium. The lower chamber was filled with 600 μL of complete medium containing 10% fetal bovine serum as a chemoattractant. After incubation for 16–24 h (migration) or 24–48 h (invasion), cells that had migrated or invaded to the lower surface were fixed with 4% paraformaldehyde for 20–30 min, stained with 0.1% crystal violet for 15 min, and counted in five randomly selected fields per well under a light microscope (200 × magnification) [39]. For both migration and invasion assays, each experiment was performed with three biological replicates per condition (i.e., three independent wells). The entire assay was repeated three independent times (on different days with different cell passages) to ensure reproducibility.

### Animal and foot pad popliteal lymph node metastasis model

All animal experiments were approved by the Institutional Ethics Committee for Animal Studies of The First Affiliated Hospital of Sun Yat-sen University. Female BALB/c nude mice (4–6 weeks old, 18–20 g) were purchased from the Animal Experiment Center (North Campus), The First Affiliated Hospital of Sun Yat-sen University, and housed under specific pathogen-free (SPF) conditions with a 12-h light/dark cycle and free access to food and water.

To establish stable FDX1-overexpressing and control cell lines, MGC803 cells were transfected with the corresponding plasmids. For the footpad popliteal lymph node metastasis model, mice were randomly assigned to experimental groups using a random number table, with 10 mice per group (control and FDX1-overexpressing groups), based on a sample size determined by power analysis (expected effect size = 0.8, α = 0.05, power = 0.8). Cells (1 × 10⁶ cells in 50 μL PBS) were injected into the left hind footpad of each mouse using a 1 mL insulin syringe.

Mice were monitored every three days for tumor growth and general health. Six weeks after injection, all mice were euthanized by cervical dislocation under isoflurane anesthesia. Popliteal lymph nodes were collected by dissection, weighed for quality assessment, and subjected to hematoxylin and eosin (H&E) staining to evaluate the presence of metastasis. All outcome assessments—including lymph node dissection, histological evaluation, and metastasis counting—were performed by two independent investigators blinded to group allocation, with group codes revealed only after data collection was complete. The incidence of lymph node metastasis was compared between groups using Fisher’s exact test, and quantitative data (e.g., lymph node weight, number of metastatic foci) were analyzed using one-way ANOVA with Tukey’s post hoc test.

### Data preprocessing

#### Log2 transformation

For datasets that were not already log2-transformed (e.g., raw count data from TCGA), we applied log2 (TPM + 1) to stabilize variance and make the data more suitable for linear modeling. This is a standard transformation in transcriptomic analyses and is known to compress extreme values but does not alter relative expression differences.

#### Quantile normalization

For datasets that had not been previously normalized, we applied quantile normalization using the normalize.quantiles function from the preprocessCore package. Quantile normalization forces the distribution of expression values to be identical across samples, which removes technical variation but may also reduce some biological variability. We acknowledge this limitation. However, we applied quantile normalization only to datasets that lacked between-sample normalization, and we did so to enable meaningful cross-sample comparisons within each dataset. For datasets that were already normalized by the original submitters, we did not apply additional quantile normalization.

#### Batch effect removal

For combined analysis of multiple datasets or multiple platforms within a dataset, we used the removeBatchEffect function from the limma package. This step adjusts for known batch covariates while preserving biological variation of interest.

### Statistical analyses

All statistical analyses were performed using R software (version 4.0.3) and GraphPad Prism 9.0. Data are presented as mean ± SD. Comparisons between two groups were performed using two-tailed Student’s t-test, while comparisons among multiple groups were assessed using one-way ANOVA followed by Tukey’s post hoc test. For transcriptomic analyses, adjusted p-values were calculated using the Benjamini–Hochberg method to control for multiple hypothesis testing; genes with an adjusted p-value < 0.05 and |log2 fold change|> 1 were considered statistically significant. In all analyses, a p-value < 0.05 was considered statistically significant. Each dataset was processed independently from its raw data files using the identical standardized pipeline. No intermediate matrices or preprocessed results were shared between datasets. The R code used for processing was the same for all datasets, but the computations were performed separately on each dataset to ensure full independence.

## Results

### Identification of cuproptosis subtypes in GCLM

**Fig. 1:**
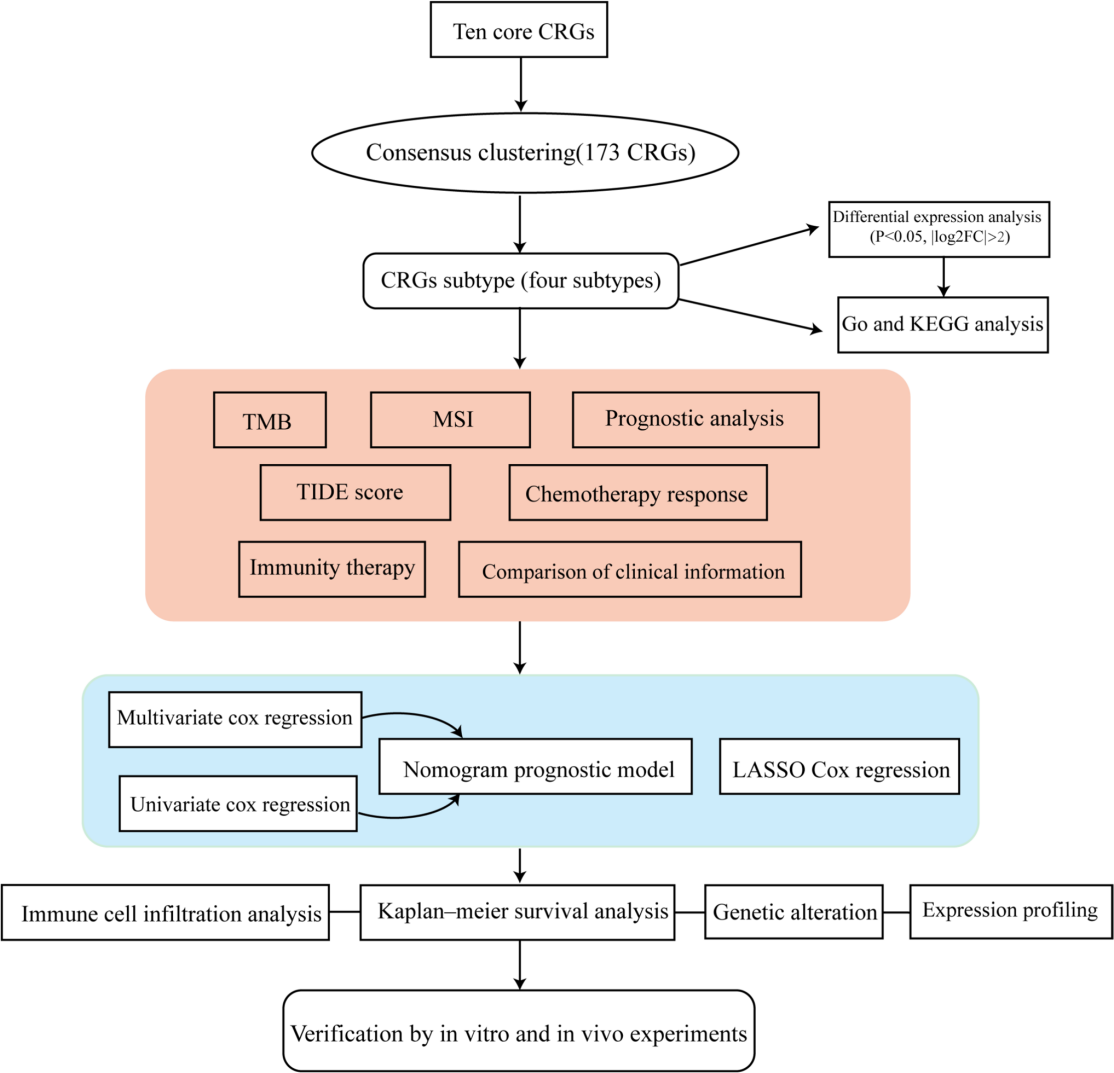
Methodological workflow of the present study

**Fig. 2:**
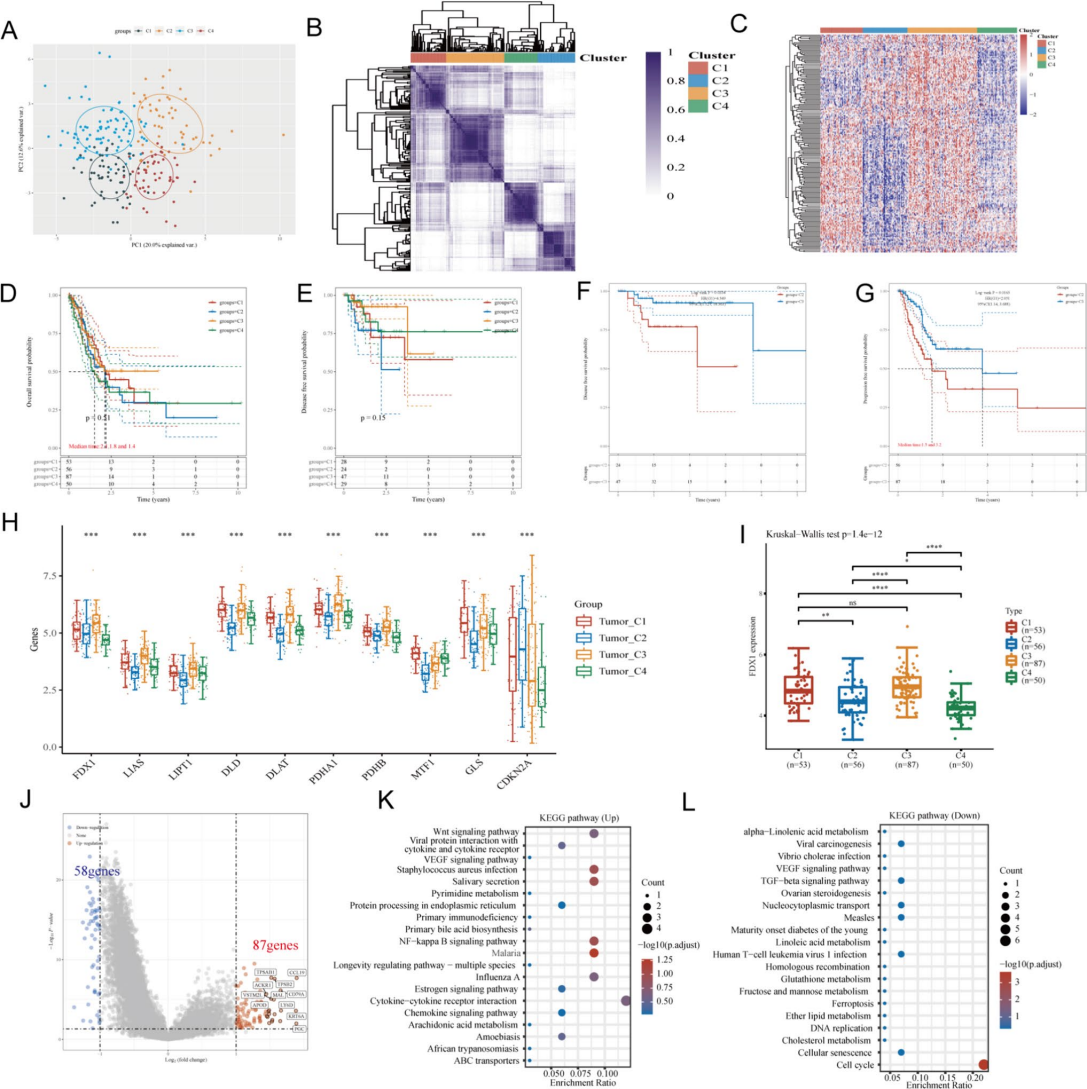
Identification of cuproptosis subtypes in GCLM. (**A**) PCA analysis showing a remarkable difference in transcriptomes among the four subtypes. (**B**) Consensus matrix heatmap defining four clusters and their correlation area. (**C**) The heatmap shows differences in expression levels of CRGs between the four distinct subtypes. (**D**–**G**) Univariate analysis showing 173 PRGs related to disease-free survival and progression-free survival times. (**H**) Expression of the 10 hub CRGs in the four subtypes. (**I**) Expression of FDX1 in the four subtypes. (**J**) An overview of the differential gene expression between the two cuproptosis-related clusters in TCGA cohorts. (**K**-**L**) KEGG enrichment analysis between the C2 and C3 subtypes. All data are presented as mean ± SEM, with significance levels indicated as *p < 0.05, **p < 0.01, ***p < 0.001, ****p < 0.0001, and ns for not significant. Each dataset was processed independently from raw data using the same standardized pipeline. Similar visual patterns reflect true biological reproducibility, not shared intermediate data

The methodological workflow of the present study is illustrated in Fig. 1. Using the expression profiles of 173 cuproptosis-related genes (CRGs) derived from a previous study [6], where these genes were identified based on the intersection of two structurally distinct copper-loaded ionophores (elesclomol and the active form of disulfiram, diethyldithiocarbamate) and filtered using a significance threshold of FDR < 0.01, we performed consensus clustering to stratify patients with GCLM (pN stage > 0) (Table S1 and Table S2). The results showed that k = 4 was the most effective selection criteria for classifying the entire cohort into subtypes C1 (n = 53), C2 (n = 56), C3 (n = 86), and C4 (n = 50) **(**Fig. 2A-C). Additionally, patients with subtype C3 had a longer DFS (p = 0.0334) and PFS (p = 0.0165) than patients with subtype C2 **(**Fig. 2D-G). Moreover, ten hub CRGs, including FDX1, DLD, LIAS, DLAT, LIPT1, PDHA1, PDHB, MTF1,GLS, and CDKN2A, were significantly different expressed in four clusters **(**Fig. 2H-I). Differential expression analysis between cuproptosis-related clusters in the TCGA cohort further highlighted subtype-specific gene expression profiles (Fig. 2J and Table S3). Additionally, the analysis conducted using the Kyoto Encyclopedia of Genes and Genomes (KEGG) highlighted a noteworthy enrichment of pathways associated with tumor immunity, inflammation, and metabolism in the context of CRGs. This finding signifies the fundamental involvement of cuproptosis in the promotion of GC proliferation. Therefore, it emphasizes the critical role played by cuproptosis in influencing various key biological processes such as tumor immunity, inflammation, and metabolism in GC (Fig. 2K-L).

### Subgroups of CRGs with different immune characteristics and mutation profiles in GCLM

**Fig. 3:**
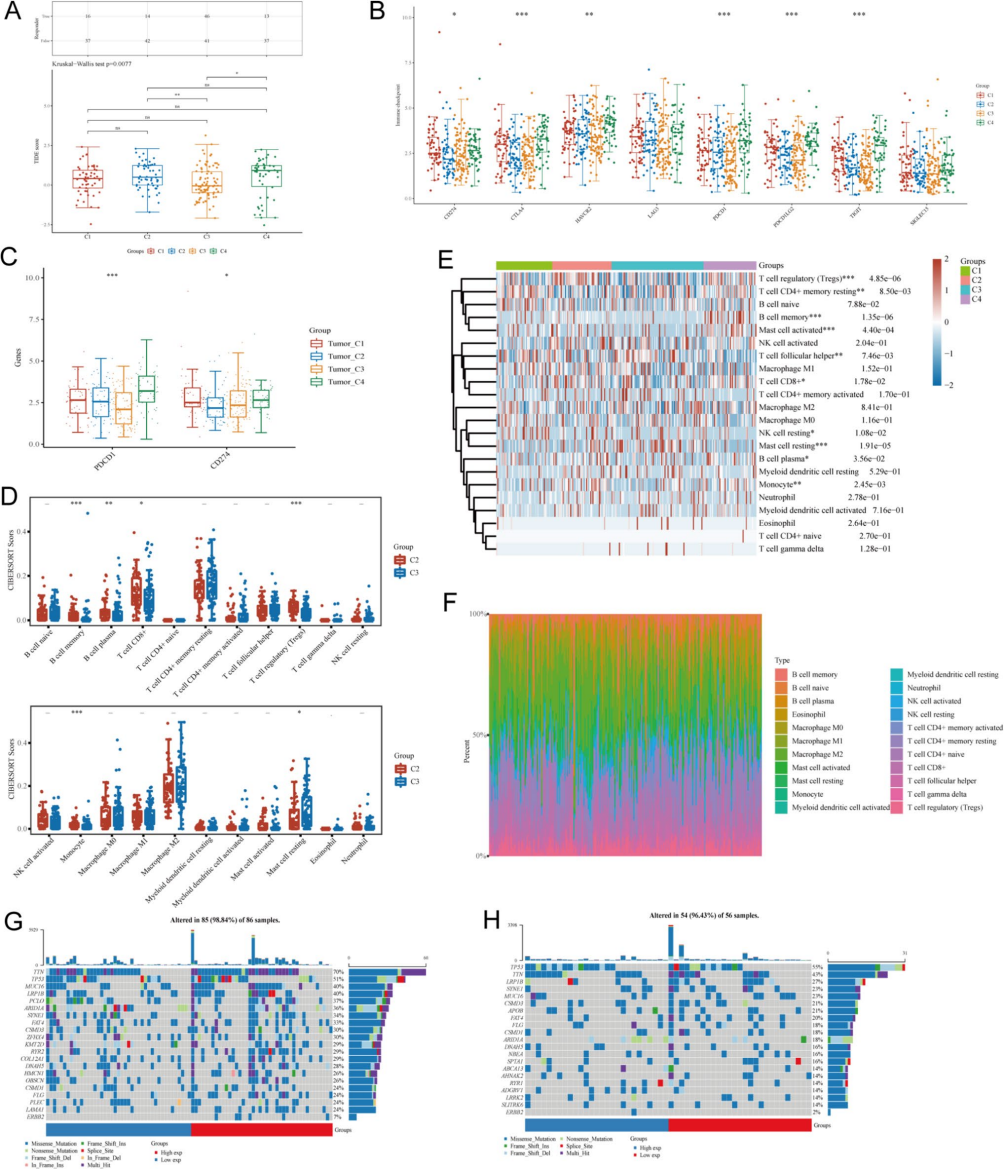
Subgroups of CRGs with different immune characteristics in GCLM. (**A**) TIDE score between different subtypes. (**B**) Expression of different checkpoint-related genes in the four subtypes. (**C**) Expression of PD1 and PD-L1 in the four subtypes. (**D**) The abundance of 22 infiltrating immune cell types in the two GC subtypes. (**E**–**F**) The heatmap abundance of the 22 infiltrating immune cell types in the two GC subtypes. (**G**-**H**)The landscape of mutation profiles in two subtypes. Data are presented as mean ± SEM. *p < 0.05; **p < 0.01; ***p < 0.001; ns p > 0.05. Each dataset was processed independently from raw data using the same standardized pipeline. Similar visual patterns reflect true biological reproducibility, not shared intermediate data

The evaluation of TIDE scores, which serves as an indicator of response to immune checkpoint blockade (ICB) and survival duration post-ICB treatment, unveiled higher scores for patients with subtype C2 (Fig. 3A). Moreover, subtype C2 demonstrated elevated levels of PD-1 expression and concurrently lower levels of PD-L1 expression (Fig. 3B-C). There was a significant difference between the two subtypes in the infiltration of immune cells with a different survival rate. Compared to the C3 subtype, the C2 subtype had higher levels of B cell memory, B cell plasma, CD8^+^ T cell, and T cell regulatory (Treg) cells. Subtype C2 exhibited significantly lower infiltration of resting mast cells in comparison to subtype C3 (Fig. 3D-F). These findings imply a diminished response to ICB therapy and reduced survival rates associated with subtype C2. We also compared the TMB of the two subtypes and found a significant difference in the proportion of mutated genes. Among them, the top 10 genes in the C2 group with the mutation ratio were TTN, TP53, MUC16, LRP1B, PCLO, ARID1A, SYNE1, FAT4, CSMD3 and ZFHX4; while the C3 group are TP53, TTN, LRP1B, SYNE1, MUC16, CSMD3, APOB, FAT4, FLG and CSMD1 (Fig. 3G and H).

### CRG subtype predicted chemotherapy response

**Fig. 4:**
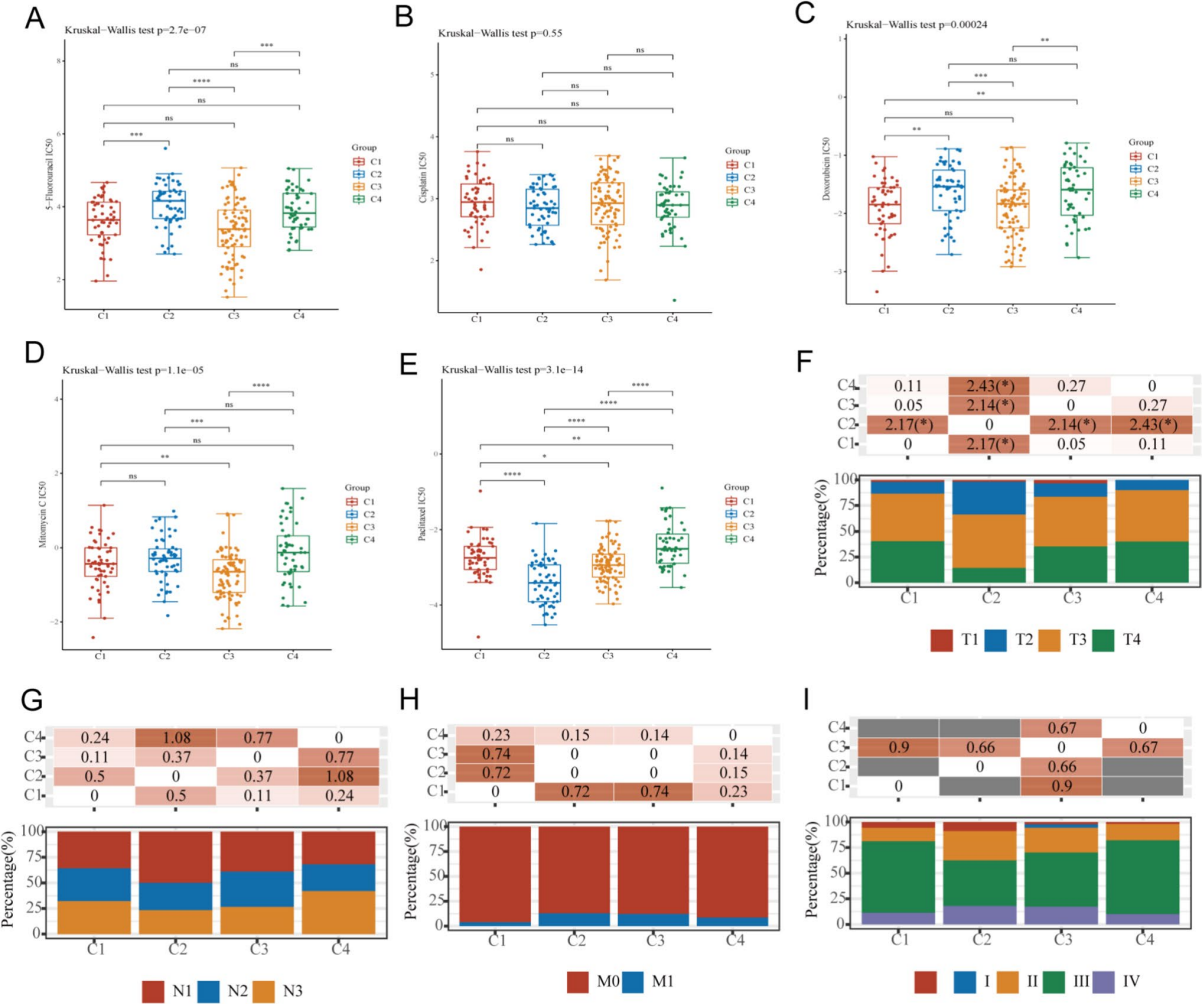
CRG subtype predicted chemotherapy response. (**A**–**E**) Associations between the four subtypes and chemotherapy sensitivity. (**F**–**I**) Comparison of clinical characteristics among the four subtypes in the TCGA cohort. Data are presented as mean ± SEM. *p < 0.05; **p < 0.01; ***p < 0.001; ****p < 0.0001; ns p > 0.05. Each dataset was processed independently from raw data using the same standardized pipeline. Similar visual patterns reflect true biological reproducibility, not shared intermediate data

To investigate the association between cancer-related genes (CRGs) and drug resistance, we developed a novel resistance score based on IC50 values, which serve as a reliable indicator of drug sensitivity in tumor cells. These IC50 values were derived from dose–response data obtained from the TCGA database, encompassing five commonly used chemotherapeutic agents: 5-fluorouracil (5-FU), doxorubicin, mitomycin, paclitaxel, and cisplatin. By applying this integrative approach, we identified significant correlations between CRGs and the IC50 values of 5-FU, doxorubicin, mitomycin, and paclitaxel, suggesting that these genes may play a crucial role in modulating chemotherapeutic sensitivity. Notably, the observed associations highlight the potential of CRGs as biomarkers for predicting drug response and as promising targets for overcoming chemoresistance in gastric cancer (Fig. 4).

### Construction of LASSO and Nomograms prognostic model

In this study, ten candidate CRGs were selected selected to construct a prognostic model using LASSO regression. Subsequent analysis led to the identification of several key factors namely FDX1, LIAS, LIPT1, MTF1, and GLS, which exhibited a significant association with the overall survival (OS) of patients diagnosed with gastric cancer (GC). Utilizing the formulated equation, a risk score was systematically calculated:

Risk score = (-0.2169) × FDX1 + (-0.0994) × LIAS + (0.0367) × LIPT1 + (-0.2555) × MTF1 + (0.0546) × GLS.

**Fig. 5:**
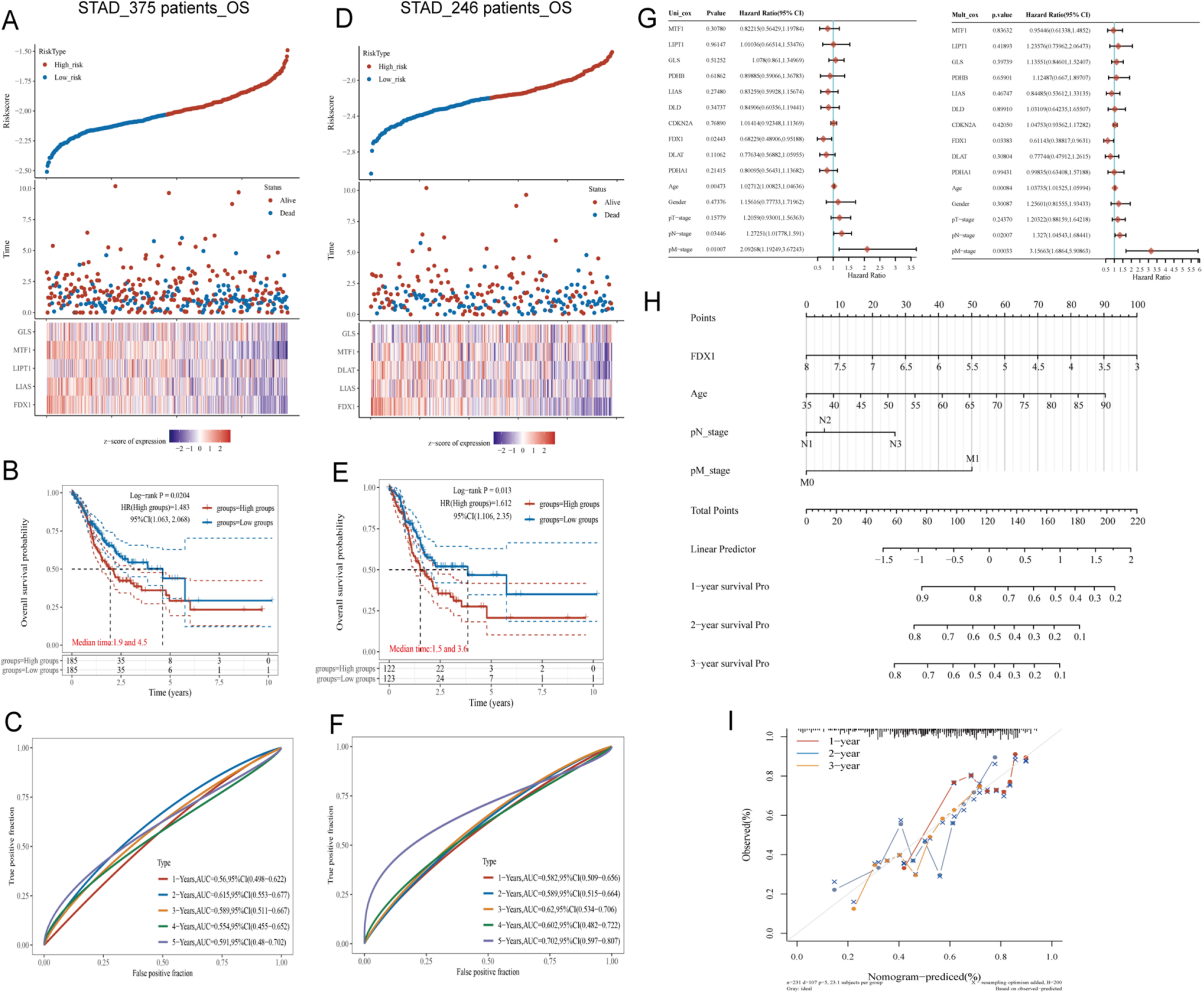
Construction of LASSO and Nomograms prognostic model. (**A** & **D**) Relationships among survival status, risk score rank, and CRG expression. (**B** & **E**) Kaplan–Meier survival curves for the high-risk and low-risk groups. (**C** & **F**) Time-dependent ROC curves evaluating the prognostic performance of the survival model in GC patients. (**G**) Forest plot showing hazard ratios (HRs) for survival-associated CRGs and clinical risk factors in GC. (**H**) Nomogram for predicting 1-, 2-, and 3-year overall survival in GC patients. (**I**) Calibration curve for the nomogram model in the training cohort. The dashed diagonal line represents the ideal prediction, while the brown, blue, orange, and purple lines represent the observed performance at 1, 2, and 3 years, respectively. Each dataset was processed independently from raw data using the same standardized pipeline. Similar visual patterns reflect true biological reproducibility, not shared intermediate data

As a result of our analysis, areas under the ROC curve (AUCs) for 1, 2, 3, 4, and 5 years were 0.56, 0.615, 0.589, 0.544, and 0.591, respectively (Fig. 5A-C). Additionally, GC patients with lymph node metastases (GCLM) had high survival rates when FDX1,LIAS, DLAT, MTF1, and GLS were present. The risk score was calculated using the following formula:

Risk score = (-0.3975) × FDX1 + (-0.0595) × LIAS + (-0.007) × DLAT + (-0.2266) × MTF1 + (0.1807) × GLS. Table 1.

**Table 1:** Clinicopathological characteristics of GCLM subtypes in the TCGA database

	C1(n = 53)	C2(n = 56)	C3(n = 87)	C4(n = 50)	P_value
Gender					0.472
Female	14	22	33	17	
Male	39	34	54	33	
Race					0.079
Asian	7	7	21	9	
Black	3	0	4	0	
White	37	39	43	38	
Islander	0	0	1	0	
Tumor size					0.028
T1b	1	1	2	0	
T2	5	14	6	4	
T2b	1	4	3	1	
T3	24	29	41	25	
T4	5	3	12	4	
T4a	12	3	9	9	
T4b	4	2	9	7	
TX	1	0	2	0	
T1	0	0	1	0	
T2a	0	0	2	0	
Lymph node metastasis					0.212
N1	19	28	34	16	
N2	17	15	30	13	
N3	4	8	8	6	
N3a	13	5	11	13	
N3b	0	0	4	2	
Distant metastasis					0.445
M0	50	48	72	43	
M1	2	7	10	4	
TNM stage					0.008
II	1	8	3	1	
IIB	6	6	17	7	
IIIA	14	16	18	9	
IIIB	14	7	14	15	
IIIC	9	2	12	12	
IV	6	10	15	5	
IIA	0	2	1	0	
I	0	0	1	0	
IB	0	0	2	0	
III	0	0	2	0	
Disease Status					0.762
Metastasis	12	13	9	8	
Metastasis:Recurrence	1	1	1		
Recurrence	8	4	4	4	
Primary	0	1	0	2	
Recurrence:Primary	0	0	0	1	
Radiation					0.422
Non-radiation	20	15	40	16	
Radiation	11	3	14	9	

As determined by the results, the AUC values for 1, 2, 3, 4, and 5 years were 0.582, 0.589, 0.62, 0.602, and 0.702 (Fig. 5D-F). According to our results, the multivariate and univariate analyses revealed that age (hazard ratio, HR = 1.03735), pN stage (HR = 1.327), and pM stage (HR = 3.15663) were prognostic variables for GCLM, while FDX1 (HR = 0.61143) acted as a protective factor (Fig. 5G). In addition, the prognostic model predicted significantly different 1-, 2-, and 3-year survival rates (C-index: 0.64, *p* < 0.001; Fig. 5H-I). These findings suggest that while the prognostic signature derived from CRGs exhibits only modest predictive accuracy in unselected gastric cancer patients, its performance is notably improved in the clinically relevant subgroup of patients with lymph node metastasis.

### Association of FDX1 with the immune microenvironment, tumor mutation burden, and microsatellite instability in gastric cancer

**Fig. 6:**
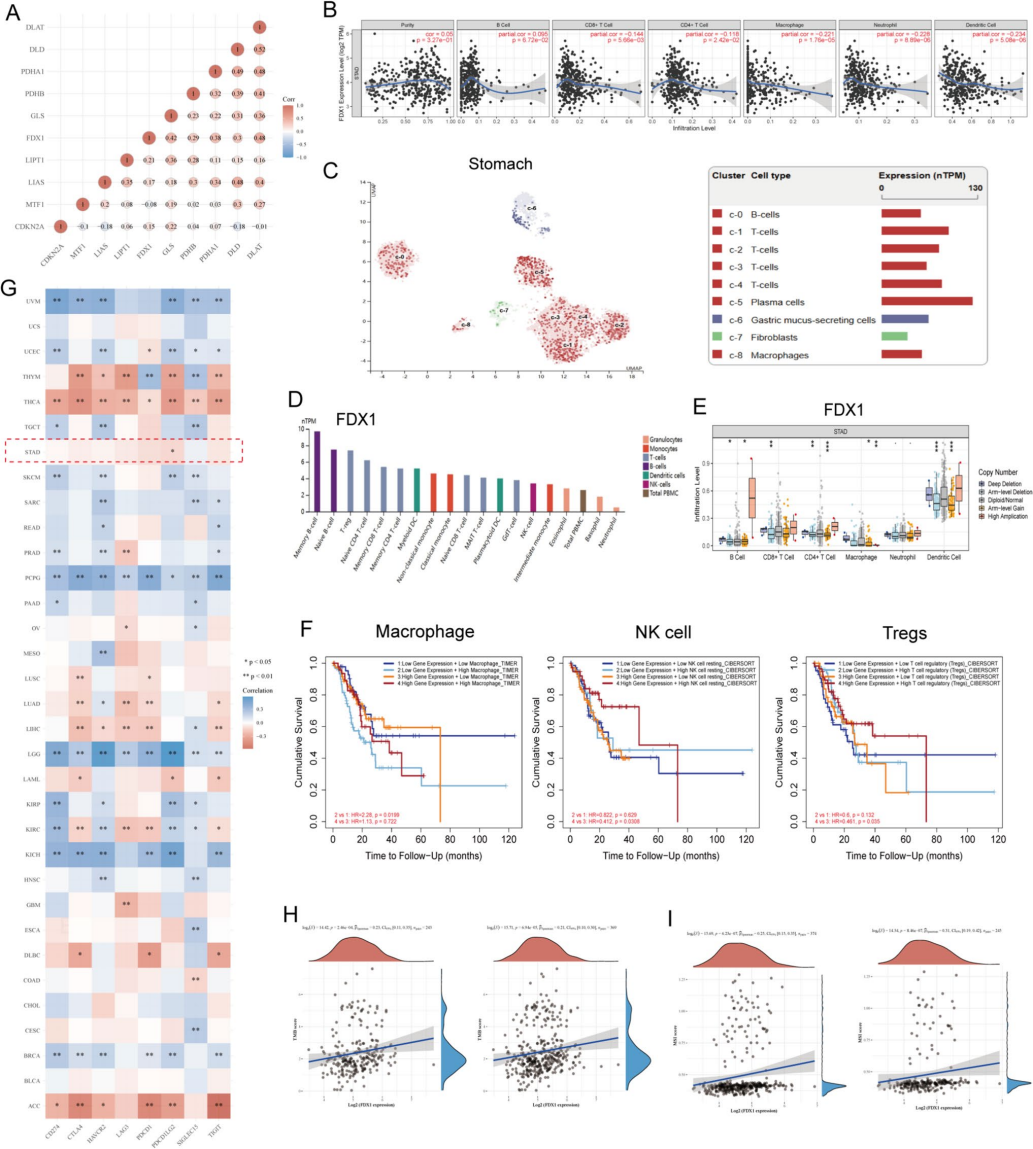
The significant interrelation between FDX1 and gastric cancer involves the immunological microenvironment and tumor mutations. (**A**) The correlation analysis reveals the interconnections among the ten cuproptosis-related genes. (**B**) The correlation between FDX1 expression level and the infiltration of immune cells. (**C**) The single-cell data in TCGA demonstrates the expression levels of FDX1 in different subpopulations of cells within stomach tissue. (**D**) The enrichment of FDX1 in different immune cells through The Human Protein Atlas database. (**E**) The correlation between FDX1 expression level and the infiltration of immune cells with different Somatic copy number alteration (sCNA) statuses. (**F**) The infiltration level is divided into low and high levels, and the hazard ratio and p value for Cox model and the log-rank p value for KM curve were shown on the KM curve plot (TIMER2.0). (**G**) Analysis of pan-cancer immune checkpoints indicates a negative correlation between FDX1 expression and PDCD1LG2 (PD-L2). (**H**–**I**) Correlations between FDX1 expression level and TMB/MSI. MSI, microsatellite instability; TMB, tumor mutation burden. Data are presented as mean ± SEM. *p < 0.05; **p < 0.01; ***p < 0.001

In our prior investigation utilizing immune scoring, we established a close association between CRGs and immune cell infiltration, as well as immune checkpoint expression levels in GC. Subsequently, by developing lasso and nomogram prognostic models, in conjunction with conducting univariate and multivariate analyses, we identified FDX1 as a prognostic factor for patients with GC. Notably, our construction of the lasso model unveiled a significant downregulation of another CRGs, DLAT, in cases of GC with lymph node metastasis, mirroring the role of FDX1 as a protective factor. Furthermore, our additional correlation analysis revealed a positive correlation (coefficient of 0.48) between FDX1 and DLAT, indicating a synergistic protective effect between these two genes (Fig. 6A). To elucidate the role of FDX1 in the tumor immune microenvironment (TIME), the TIMER databases were used to analyze immune cell infiltration. As a result, a negative correlation was observed between expression of FDX1 and CD4^+^ T cells infiltration (cor =  − 0.118, *p* = 2.42e − 02), CD8 + T cell (cor =  − 0.144, *p* = 5.66e − 03), macrophages (cor =  − 0.221, *p* = 1.76e − 05), neutrophils (cor =  − 0.228, *p* = 8.89e − 06), and dendritic cells infiltration (DCs; cor =  − 0.234, *p* = 5.08e − 06) (Fig. 6B, Figs. S1^2^,3,4,5). Through single-cell analysis in the human protein atlas database, it has been revealed that there are notably elevated expression levels of FDX1 in T cells, B cells, and gastric glandular epithelial cells (Fig. 6C-D). Meanwhile, we compared immune infiltration distribution using the sCNA status of candidate genes across STAD samples from the TCGA database. In addition, we analyzed the enrichment of hub CRGs in different immune cells through the TCGA database and found that FDX1 were mainly enriched in memory B cells, naive CD4^+^ T cells, non-classical monocytes, and malt T cells, respectively (Fig. 6E, Fig. S6). Moreover, the COX regression model analysis of the immune cell infiltration score revealed that higher macrophage infiltration were significantly associated with the poor prognosis of GC patients having lower FDX1 expression levels, while lower NK cell and Tregs infiltration were related to poor prognosis of GC patients having higher FDX1 expression (Fig. 6F). Additionally, a significant correlation was also observed between FDX1 expression levels and TMB and microsatellite instability (MSI) (Fig. 6H-I). We performed exploratory correlation analyses between FDX1 expression and a panel of immune checkpoint genes (e.g., PD-1, PD-L1, CTLA-4) in the TCGA-STAD cohort. Additionally, the Tumor Immune Dysfunction and Exclusion (TIDE) score was applied to predict the response to immune checkpoint inhibitors based on FDX1 expression levels in gastric cancer. As shown in Fig. S7, FDX1 expression was negatively correlated with multiple immune checkpoint genes, and patients with high FDX1 expression exhibited a significantly poorer response to immunotherapy, suggesting that tumors with elevated FDX1 expression may display diminished immune activation signals. Although preliminary, these findings provide a potential link between FDX1 expression and the “cold” immune phenotype observed in our study. Consistent with this, the favorable prognosis associated with high FDX1 expression appears to be primarily driven by tumor-intrinsic mechanisms, such as reduced metastatic potential, rather than by antitumor immunity.

### FDX is correlated with a favorable prognosis and serves to inhibit tumor metastasis in GC patients

**Fig. 7:**
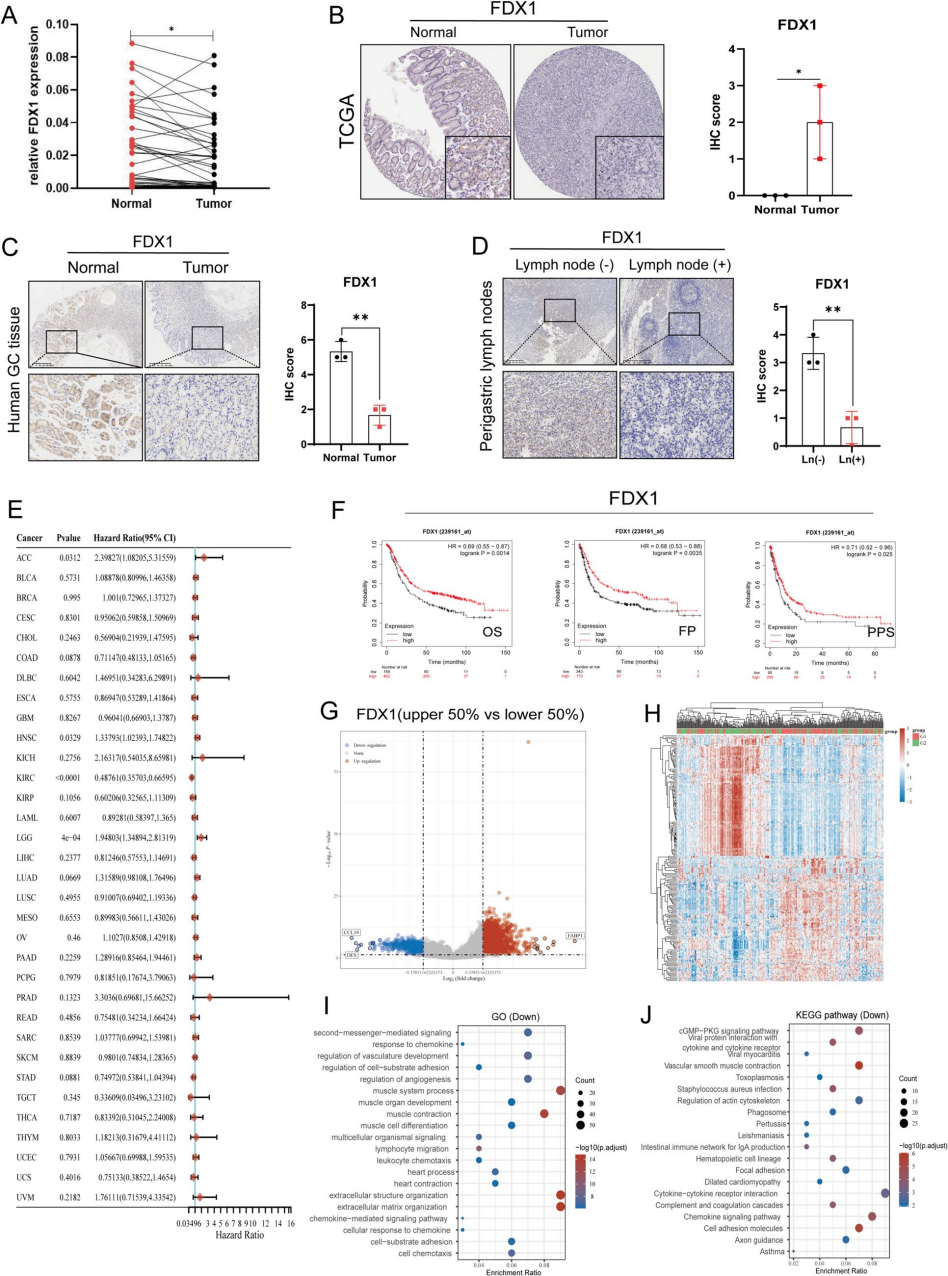
FDX is correlated with a favorable prognosis and serves to inhibit tumor metastasis in GC patients. (**A**) qPCR results revealed that FDX1 was low-expressed in GC tissues. (**B**) TCGA gastric cancer immunohistochemical data showed FDX1 was lowly expressed in GC tissues. (**C**-**D**) Expression of FDX1 in adjacent, primary cancer, and negative and positive lymph nodes determined by IHC. (**E**) Randomized forest plots illustrated that FDX1 was significantly related to the favorable prognosis of GC. (**F**) The Kaplan–Meier plotter database was used to plot overall survival, FP, and post-progression survival curves at a threshold of *p* < 0.05 to compare patients with high (red) and low (black) expressions of FDX1in GC patients. (**G**-**H**) The volcano plot and heatmap illustrated the differential gene distribution of high and low expression of FDX1 in GC patients. (**I**-**J**) GO and KEGG enrichment analysis between the high and low expression of FDX1 groups. Data are presented as mean ± SEM. *p < 0.05; **p < 0.01

To assess the expression of the FDX1 in both normal and tumor tissues in GC, our study employed data from the TCGA database, quantitative Polymerase Chain Reaction (qPCR), and Immunohistochemistry (IHC) staining. This analysis aimed to investigate potential correlations between FDX1 expression levels and GC stages and associated outcomes. We examined the expression of FDX1 mRNA in clinical samples, and the results showed low FDX1 expression in tumor tissues (Fig. 7A). Furthermore, the IHC results suggest a notable reduction in the expression levels of FDX1 within GC tissue (Fig. 7B). Moreover, IHC staining revealed low FDX1 expression in primary GCs and metastatic lymph nodes, while high FDX1 expression was found in adjacent and non-metastatic lymph nodes (Fig. 7C-D). Through survival analysis of external GEO datasets, we found that high FDX1 expression was significantly associated with favorable prognosis in gastric cancer. In addition, we performed survival analyses for the remaining nine hub cuproptosis-related genes and validated their prognostic significance in gastric cancer. In patients with gastric cancer, higher FDX1 expression was associated with longer overall survival (OS), progression-free survival (PFS), and post-progression survival (PPS), indicating that FDX1 expression levels correlate with favorable prognosis in gastric cancer (Fig. 7E-F and Fig. S8). To investigate the potential impact mechanism of FDX1 expression on lymph node metastasis in GC, we conducted a comparative analysis between high and low FDX1 expression cases using the TCGA database (Fig. 7G-H). Our findings indicate that high FDX1 expression suppresses the metastatic capability of gastric cancer. Furthermore, GO and KEGG enrichment analyses revealed that pathways and biological processes associated with tumor metastasis—including focal adhesion, cell adhesion molecules, regulation of the actin cytoskeleton, regulation of angiogenesis, and extracellular matrix organization—were significantly downregulated in gastric cancer tissues (Fig. 7I-J).

### Upregulation of FDX1 significantly inhibits lymph node metastasis in GC

**Fig. 8:**
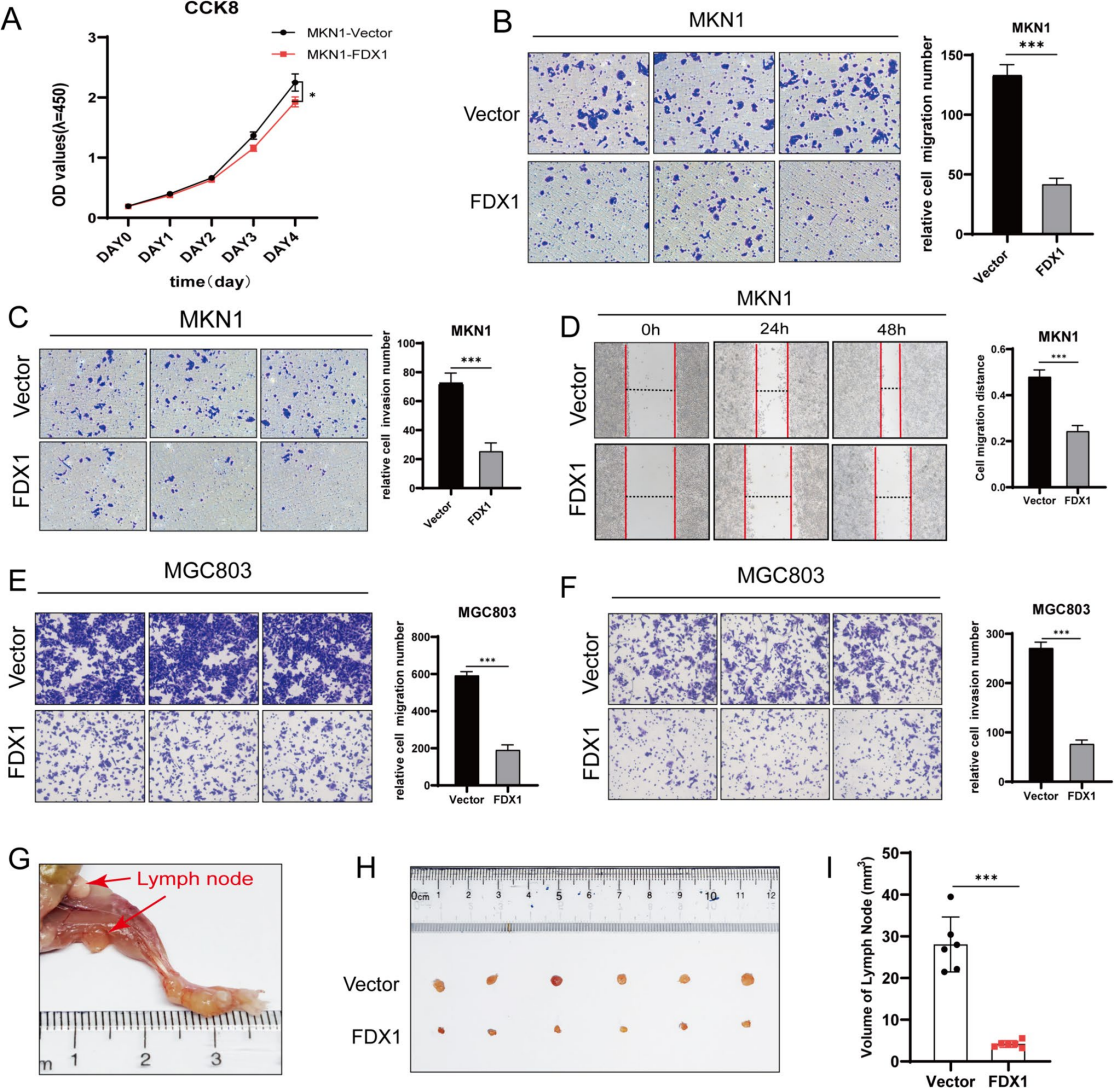
Upregulation of FDX1 significantly inhibits lymph node metastasis in GC. (**A**) Experiments on CCK8 showed that FDX1 overexpression inhibits the proliferation of GC cells. (**B**-**D**) Transwell and scratch assay displaying FDX1 overexpression suppressed invasion and migration in MKN1 cells. (**E**–**F**) Transwell assay displaying FDX1 overexpression suppressed invasion and migration in MGC803 cells. (**G**-**I**) Mouse popliteal lymph node metastasis model showing the ability of lymph node metastasis was significantly diminished upregulation of FDX1. The exact number of animals per group (n = 10), the randomization method (random number table), and the blinding procedures (blinded assessment of all outcomes by two independent investigators). G: popliteal lymph node metastasis anatomy and lymph node tissue, H&I: lymph node volume contrast histogram. Data are presented as mean ± SEM. *p < 0.05; ***p < 0.001

To further investigate the involvement of FDX1 in tumor cell proliferation and metastasis—given the established positive correlation between lymph node involvement and metastatic potential—we established MKN1 and MGC803 gastric cancer cell lines with stable FDX1 overexpression via plasmid transfection. Functional assays, including CCK8 proliferation assays as well as Transwell migration and invasion assays, demonstrated that FDX1 overexpression significantly suppressed the proliferative and migratory capacities of gastric cancer cells (Fig. 8A–F). Notably, the inhibitory effect was more pronounced in metastatic functions, as evidenced by the greater reduction in migration and invasion capacities compared to proliferation. To assess the role of FDX1 in lymph node metastasis in vivo, we employed a BALB/c mouse footpad implantation model. Consistent with our in vitro findings, FDX1 overexpression resulted in a marked reduction in the lymph node metastatic capacity of gastric cancer cells (Fig. 8G–I). These results collectively suggest that FDX1 plays a critical inhibitory role in both the proliferative and metastatic behavior of gastric cancer.

## Discussion

In recent years, substantial progress has been made in understanding the mechanisms by which iron and other essential metals contribute to various types of human cancer. These discoveries have proven to be immensely valuable. Nevertheless, despite these advances, the precise mechanisms underlying copper-induced cytotoxicity in cancer remain largely elusive [7, 8]. The objective of this study was to investigate the mRNA and protein expression, immune response, mutational status, drug resistance, and prognostic significance of cuproptosis-related genes (CRGs) in gastric cancer with lymph node metastasis (GCLM). We further validated our findings through both in vitro and in vivo experiments. Additionally, we made significant progress in establishing a comprehensive framework to elucidate the relationships among these models, immune responses, mutational profiles, and chemotherapeutic resistance. We have successfully identified four distinct molecular subtypes using a panel of 173 Cancer-Related Genes (CRGs). Upon analysis, we discovered significant differences between two specific subtypes, namely C2 and C3, with regards to their prognostic value. Notably, patients belonging to subtype C2 exhibited a worse disease-free survival rate and displayed more advanced clinicopathological characteristics compared to those in subtype C3. Additionally, our study identified significant differences among the four gastric cancer (GC) subtypes with respect to mRNA transcriptomes, clinicopathological characteristics, prognosis, tumor mutational burden (TMB), tumor immune microenvironment (TIME), microsatellite instability (MSI), and drug sensitivity. These findings suggest that CRGs may serve as a valuable tool for predicting both prognosis and therapeutic efficacy in patients with GC.

The research conducted by Tsvetkov et al. elucidated the regulatory mechanisms underlying copper-induced cell death and identified several critical regulators, including FDX1, LIAS, LIPT1, DLD, DLAT, PDHA1, PDHB, MTF1, and CDKN2A [6]. These regulators have been identified as essential factors in governing cellular responses to copper-related cytotoxicity. Such comprehensive analysis underscores their potential significance in understanding the molecular pathways involved in copper-induced cell death [6]. Currently, immunotherapy has become indispensable in tumor treatment, and exploring the interdependence between infiltrating immune cells and malignant tumors is necessary [40]. Lymph node metastasis has been identified as the primary avenue for gastric cancer metastasis. Our study revealed that the expression of CRGs plays a crucial role in determining the prognosis of patients with GCLM. Notably, our findings elucidated the significant prognostic value of CRGs in GCLM patients. A subclass analysis provided additional insights into the significant differences in disease-free survival observed between two distinct groups of patients. These groups exhibited contrasting clinical characteristics, immune cell infiltration patterns, ICB scores, tumor mutational burden (TMB), as well as varying levels of resistance to chemotherapeutic drugs. Subsequently, a multivariate regression analysis identified FDX1 as a potential independent prognostic factor for GC. In tumors, FDX1 encodes a protein that reduces Cu2^+^ to Cu1^+^, which is directly targeted by elesclomol [7]. Recent studies have revealed a complex, context-dependent relationship between FDX1 expression, immune infiltration, and prognosis. A pan-cancer analysis by Yang et al. [41] showed that in stomach adenocarcinoma, high FDX1 expression correlates with reduced CD8^+^ T cell infiltration yet paradoxically predicts better overall survival. Mechanistically, FDX1 primarily functions as a regulator of mitochondrial metabolism and redox balance rather than a direct immune modulator; its high expression may improve survival by reducing metastatic potential independently of antitumor immunity. Although cuproptosis can trigger immunogenic cell death [42], the relationship between FDX1 expression and cuproptosis sensitivity is non-linear [43]. Additionally, high FDX1 expression may promote immune evasion through non-canonical pathways, such as modulating immune checkpoint expression [42]. Collectively, these findings underscore the multifactorial and context-dependent interplay between FDX1, cuproptosis, and the tumor immune microenvironment. Our study found a substantial correlation between elevated FDX1 expression and overall survival (OS), disease-free survival (DFS), and post-progression survival (PPS) in patients with gastric cancer. Furthermore, we observed a significant inverse relationship between the levels of FDX1 expression and the quantities of CD8^+^ T cells, CD4^+^ T cells, macrophages, neutrophils, and dendritic cells (DCs). These findings indicate that FDX1 expression may play a crucial role in modulating the tumor microenvironment in GC.

The role of epigenetic changes in the early stages of malignant tumors has been clarified in recent years by a series of studies [44]. The elevated mutation rate of CRGs in GC likely influences the genetic characteristics of GC patients. Notably, FDX1 may play a pivotal role in the malignant progression of GC. Moreover, this research underscores a significant association between increased expression levels of FDX1 and a favorable prognosis in patients diagnosed with GCLM. These findings provide valuable insights for the advancement of new molecular classifications in the study of GC. Additionally, our GO and KEGG enrichment analyses revealed that FDX1 expression is associated with the modulation of key metastasis-related pathways, including focal adhesion, cell adhesion molecules, regulation of angiogenesis and regulation of the actin cytoskeleton. Given the established role of FDX1 in mitochondrial respiration and copper metabolism [42, 45], we further explored its potential mechanistic links to metabolic pathways and oxidative stress. Recent studies have shown that FDX1, as a key regulator of mitochondrial oxidative phosphorylation, can influence endothelial cell function and angiogenic signaling through modulation of reactive oxygen species (ROS) and metabolic intermediates [46]. In gastric cancer, mitochondrial uncoupling has been shown to remodel cellular metabolism and enhance cuproptosis sensitivity through the upregulation of FDX1 expression [47]. These findings suggest that FDX1 may influence the malignant behavior of gastric cancer cells by modulating mitochondrial metabolism and redox homeostasis. However, we acknowledge that these proposed mechanisms remain speculative at this stage. Future studies—including metabolomic profiling, assessment of mitochondrial function and reactive oxygen species (ROS) levels, and investigation of copper-dependent enzyme activities in FDX1-manipulated cells—are warranted to definitively elucidate the molecular pathways underlying the anti-metastatic effects of FDX1 in gastric cancer.

Our data are consistent with the possibility that the favorable prognosis associated with high FDX1 expression is primarily driven by tumor-intrinsic mechanisms, such as reduced metastatic potential, although this interpretation remains speculative. What our findings clearly demonstrate is that FDX1 overexpression suppresses lymph node metastasis in vitro and in vivo, and that high FDX1 expression correlates with both improved survival and lower CD8⁺ T cell infiltration in public datasets. The TIME is a highly integrated system in which vascular remodeling, immune cell exclusion, and tumor-intrinsic properties collectively shape metastatic outcomes [48]. Our observation that high FDX1 expression correlates with both reduced CD8⁺ T cell infiltration and diminished metastatic capacity may therefore reflect such integrated TIME dynamics, though the causal relationships remain to be elucidated. From the current data, it is not possible to determine whether the survival benefit is causally attributable to reduced metastatic potential, reduced immune infiltration, or other unmeasured factors. The hypothesis that FDX1 acts primarily through tumor-intrinsic mechanisms-while plausible and consistent with our functional experiments—remains speculative and requires direct experimental testing.

Our study reveals that CRGs contribute to the malignant progression of gastric cancer, albeit in diverse and multifaceted ways that lack uniformity. Notably, the CRG-based signature demonstrated improved prognostic stratification specifically in patients with lymph node metastasis. However, its modest discriminatory capacity underscores the need for external validation using larger, independent cohorts. Future studies may also enhance predictive accuracy by integrating additional biomarkers, such as immune-related features or functional genomic alterations. Furthermore, additional experimental validation will be essential to strengthen the reliability of our findings. Thus, while the CRG-based prognostic signature demonstrates reproducible prognostic associations across multiple datasets, it should currently be considered an exploratory prognostic indicator rather than a clinically validated tool. External validation in a prospective, multicenter cohort is required before clinical application.

## Conclusions

In summary, this study identifies FDX1 as a potential key regulator of lymph node metastasis in gastric cancer and provides a preliminary CRG-based prognostic signature that shows reproducible associations across multiple retrospective cohorts. However, given the modest predictive performance, the model should currently be considered an exploratory tool for generating hypotheses rather than a clinically actionable instrument. Further external validation and mechanistic studies are needed to determine whether FDX1 or the CRG signature can ultimately inform clinical decision-making.

## Supplementary Information

Below is the link to the electronic supplementary material.

Supplementary file1

Supplementary file2

Supplementary file3

Supplementary file4

Supplementary file5

Supplementary file6

Supplementary file7

Supplementary file8

Supplementary file9

Supplementary file10

Supplementary file11

## Data Availability

The bulk RNA-seq data and clinical information of the TCGA cohorts and GEO cohorts were obtained from the Genomic Data Commons Data Portal (https://portal.gdc.cancer.gov/), Timer 2.0 (https://timer.cistrome.org), GEPIA2.0 (http://gepia2.cancer-pku.cn/#analysis), and Kaplan–Meier Plotter (https://kmplot.com/analysis/), respectively. All data generated in this study are available from the corresponding author upon request.
