# Supplementary material for: FDX1 as a predictive biomarker and therapeutic target for lymph node metastasis in gastric cancer
Source: Clin Exp Med. 2026 May 10;26(1):245. doi: 10.1007/s10238-026-02160-0 (PMC13331937; doi:10.1007/s10238-026-02160-0)
Supplement: Supplementary file 3 — Supplementary file3 [file 10238_2026_2160_MOESM3_ESM.pdf]

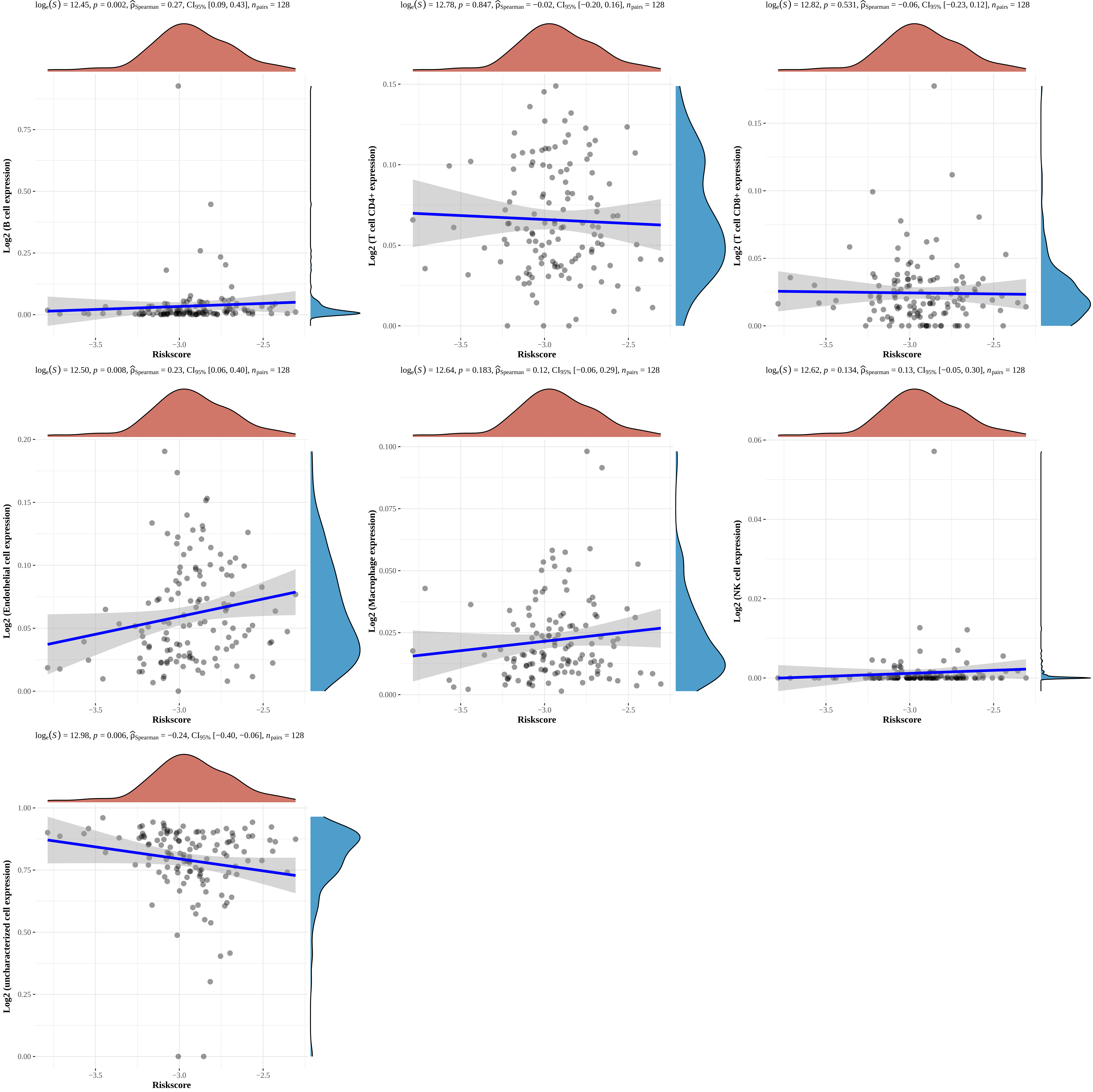

log_e(S) = 12.45, p = 0.002, ρ̂_Spearman = 0.27, CI95% [0.09, 0.43], n_pairs = 128
Log2 (B cell expression)
0.00
0.25
0.50
0.75
Riskscore
−3.5
−3.0
−2.5
log_e(S) = 12.78, p = 0.847, ρ̂_Spearman = −0.02, CI95% [−0.20, 0.16], n_pairs = 128
Log2 (T cell CD4+ expression)
0.00
0.05
0.10
0.15
Riskscore
log_e(S) = 12.82, p = 0.531, ρ̂_Spearman = −0.06, CI95% [−0.23, 0.12], n_pairs = 128
Log2 (T cell CD8+ expression)
0.00
0.05
0.10
0.15
Riskscore
log_e(S) = 12.50, p = 0.008, ρ̂_Spearman = 0.23, CI95% [0.06, 0.40], n_pairs = 128
Log2 (Endothelial cell expression)
0.00
0.05
0.10
0.15
0.20
Riskscore
log_e(S) = 12.64, p = 0.183, ρ̂_Spearman = 0.12, CI95% [−0.06, 0.29], n_pairs = 128
Log2 (Macrophage expression)
0.000
0.025
0.050
0.075
0.100
Riskscore
log_e(S) = 12.62, p = 0.134, ρ̂_Spearman = 0.13, CI95% [−0.05, 0.30], n_pairs = 128
Log2 (NK cell expression)
0.00
0.02
0.04
0.06
Riskscore
log_e(S) = 12.98, p = 0.006, ρ̂_Spearman = −0.24, CI95% [−0.40, −0.06], n_pairs = 128
Log2 (uncharacterized cell expression)
0.00
0.25
0.50
0.75
1.00
Riskscore
