# Supplementary material for: FDX1 as a predictive biomarker and therapeutic target for lymph node metastasis in gastric cancer
Source: Clin Exp Med. 2026 May 10;26(1):245. doi: 10.1007/s10238-026-02160-0 (PMC13331937; doi:10.1007/s10238-026-02160-0)
Supplement: Supplementary file 4 — Supplementary file4 [file 10238_2026_2160_MOESM4_ESM.pdf]

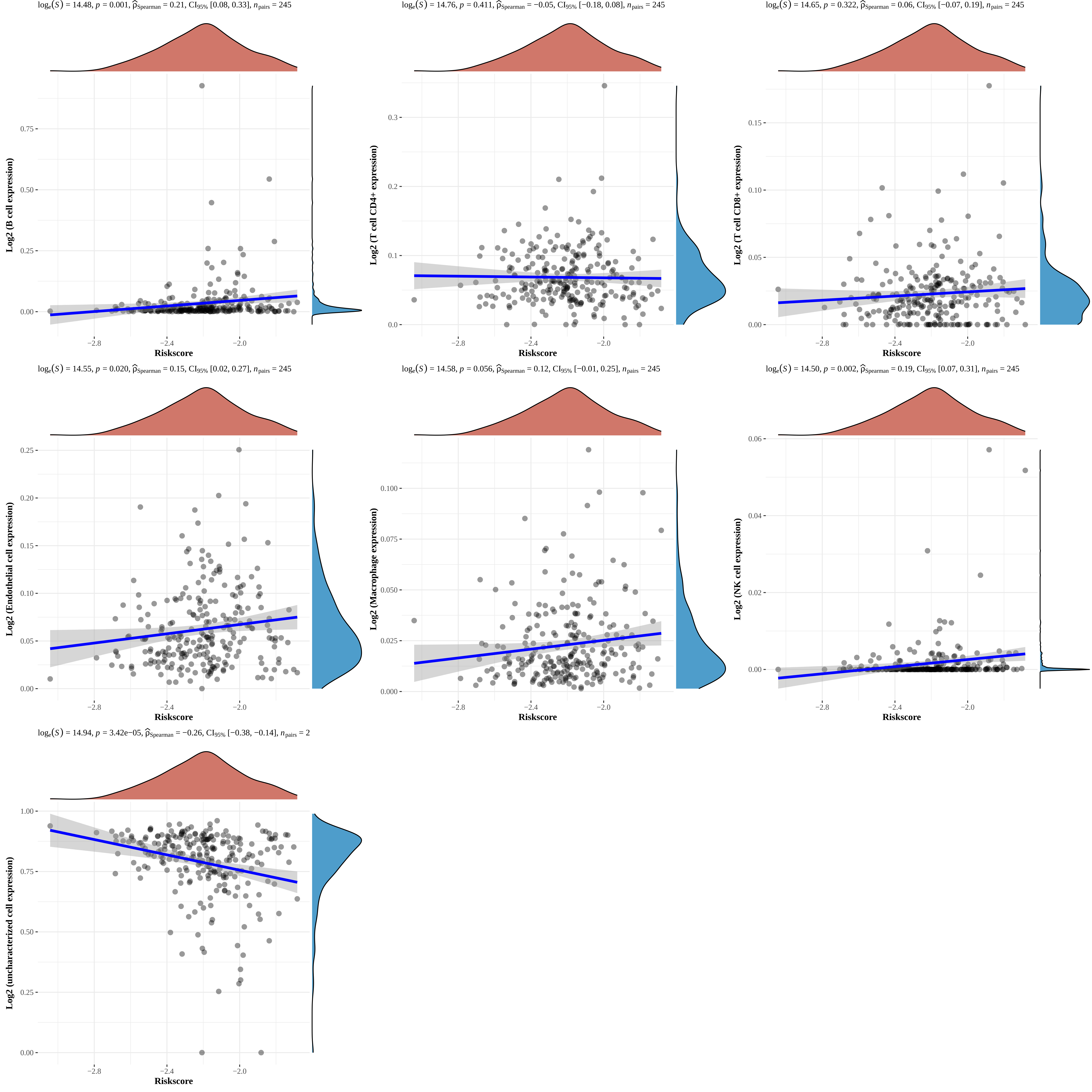

loge(S) = 14.48, p = 0.001, ρ̂Spearman = 0.21, CI95% [0.08, 0.33], npairs = 245
Log2 (B cell expression)
Riskscore
loge(S) = 14.76, p = 0.411, ρ̂Spearman = −0.05, CI95% [−0.18, 0.08], npairs = 245
Log2 (T cell CD4+ expression)
Riskscore
loge(S) = 14.65, p = 0.322, ρ̂Spearman = 0.06, CI95% [−0.07, 0.19], npairs = 245
Log2 (T cell CD8+ expression)
Riskscore
loge(S) = 14.55, p = 0.020, ρ̂Spearman = 0.15, CI95% [0.02, 0.27], npairs = 245
Log2 (Endothelial cell expression)
Riskscore
loge(S) = 14.58, p = 0.056, ρ̂Spearman = 0.12, CI95% [−0.01, 0.25], npairs = 245
Log2 (Macrophage expression)
Riskscore
loge(S) = 14.50, p = 0.002, ρ̂Spearman = 0.19, CI95% [0.07, 0.31], npairs = 245
Log2 (NK cell expression)
Riskscore
loge(S) = 14.94, p = 3.42e−05, ρ̂Spearman = −0.26, CI95% [−0.38, −0.14], npairs = 2
Log2 (uncharacterized cell expression)
Riskscore
