# Supplementary material for: FDX1 as a predictive biomarker and therapeutic target for lymph node metastasis in gastric cancer
Source: Clin Exp Med. 2026 May 10;26(1):245. doi: 10.1007/s10238-026-02160-0 (PMC13331937; doi:10.1007/s10238-026-02160-0)
Supplement: Supplementary file 5 — Supplementary file5 [file 10238_2026_2160_MOESM5_ESM.pdf]

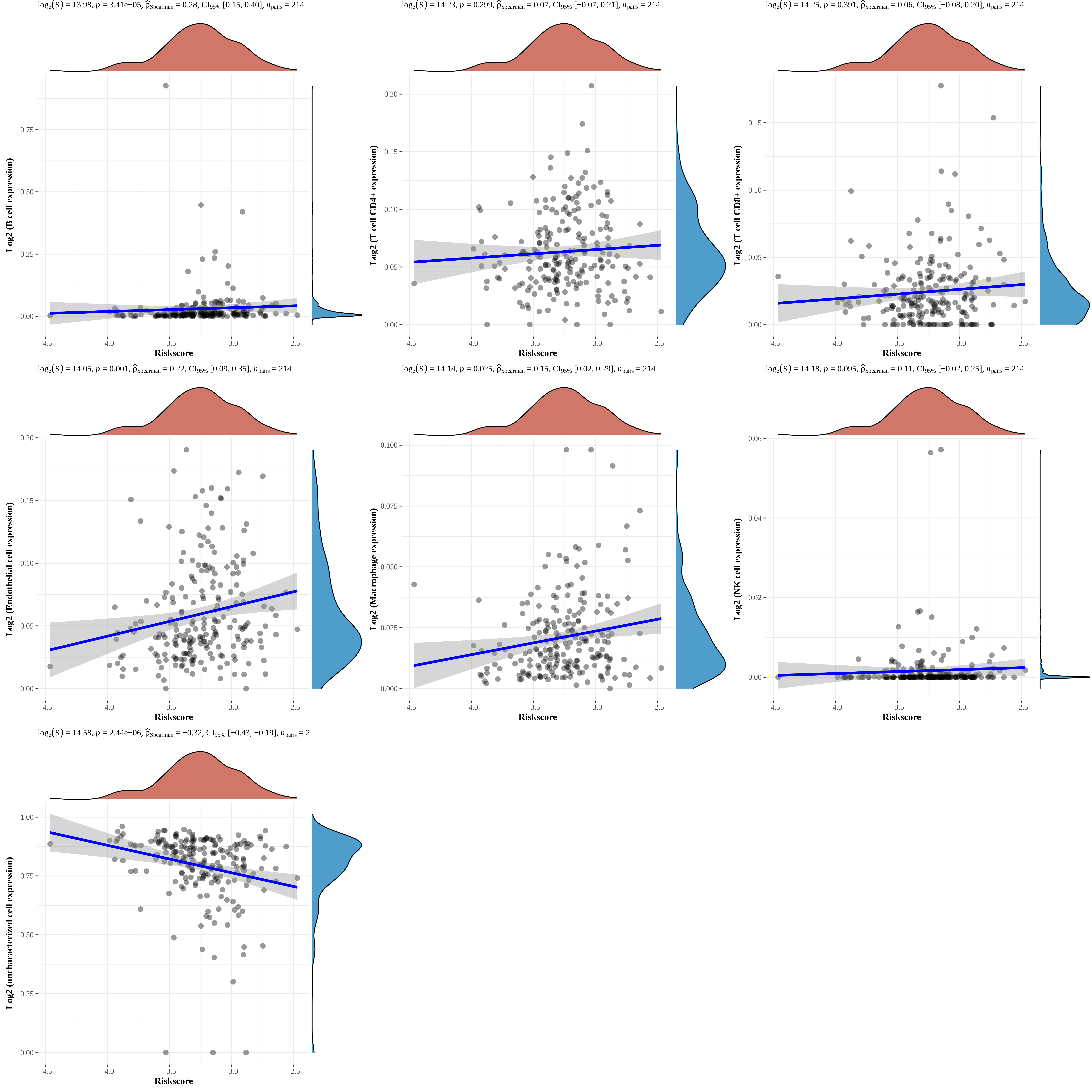

log_e(S) = 13.98, p = 3.41e−05, ρ̂_Spearman = 0.28, CI_95% [0.15, 0.40], n_pairs = 214
Log2 (B cell expression)
Riskscore
log_e(S) = 14.23, p = 0.299, ρ̂_Spearman = 0.07, CI_95% [−0.07, 0.21], n_pairs = 214
Log2 (T cell CD4+ expression)
Riskscore
log_e(S) = 14.25, p = 0.391, ρ̂_Spearman = 0.06, CI_95% [−0.08, 0.20], n_pairs = 214
Log2 (T cell CD8+ expression)
Riskscore
log_e(S) = 14.05, p = 0.001, ρ̂_Spearman = 0.22, CI_95% [0.09, 0.35], n_pairs = 214
Log2 (Endothelial cell expression)
Riskscore
log_e(S) = 14.14, p = 0.025, ρ̂_Spearman = 0.15, CI_95% [0.02, 0.29], n_pairs = 214
Log2 (Macrophage expression)
Riskscore
log_e(S) = 14.18, p = 0.095, ρ̂_Spearman = 0.11, CI_95% [−0.02, 0.25], n_pairs = 214
Log2 (NK cell expression)
Riskscore
log_e(S) = 14.58, p = 2.44e−06, ρ̂_Spearman = −0.32, CI_95% [−0.43, −0.19], n_pairs = 2
Log2 (uncharacterized cell expression)
Riskscore
