# Supplementary material for: FDX1 as a predictive biomarker and therapeutic target for lymph node metastasis in gastric cancer
Source: Clin Exp Med. 2026 May 10;26(1):245. doi: 10.1007/s10238-026-02160-0 (PMC13331937; doi:10.1007/s10238-026-02160-0)
Supplement: Supplementary file 6 — Supplementary file6 [file 10238_2026_2160_MOESM6_ESM.pdf]

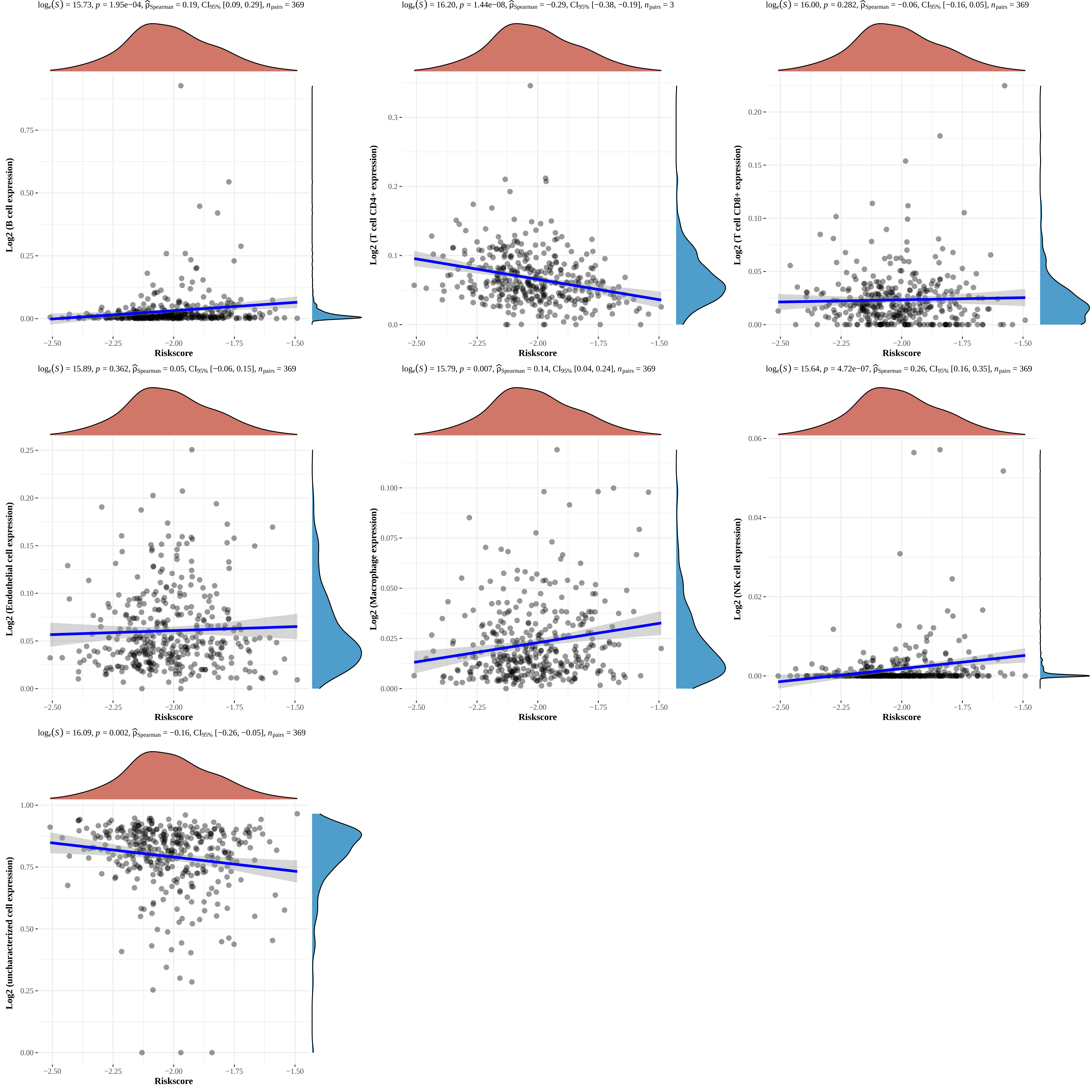

loge(S) = 15.73, p = 1.95e−04, ρ̂Spearman = 0.19, CI95% [0.09, 0.29], npairs = 369
Log2 (B cell expression)
Riskscore
loge(S) = 16.20, p = 1.44e−08, ρ̂Spearman = −0.29, CI95% [−0.38, −0.19], npairs = 3
Log2 (T cell CD4+ expression)
Riskscore
loge(S) = 16.00, p = 0.282, ρ̂Spearman = −0.06, CI95% [−0.16, 0.05], npairs = 369
Log2 (T cell CD8+ expression)
Riskscore
loge(S) = 15.89, p = 0.362, ρ̂Spearman = 0.05, CI95% [−0.06, 0.15], npairs = 369
Log2 (Endothelial cell expression)
Riskscore
loge(S) = 15.79, p = 0.007, ρ̂Spearman = 0.14, CI95% [0.04, 0.24], npairs = 369
Log2 (Macrophage expression)
Riskscore
loge(S) = 15.64, p = 4.72e−07, ρ̂Spearman = 0.26, CI95% [0.16, 0.35], npairs = 369
Log2 (NK cell expression)
Riskscore
loge(S) = 16.09, p = 0.002, ρ̂Spearman = −0.16, CI95% [−0.26, −0.05], npairs = 369
Log2 (uncharacterized cell expression)
Riskscore
