# Supplementary figures and images for: FDX1 as a predictive biomarker and therapeutic target for lymph node metastasis in gastric cancer
Source: Clin Exp Med. 2026 May 10;26(1):245. doi: 10.1007/s10238-026-02160-0 (PMC13331937; doi:10.1007/s10238-026-02160-0)

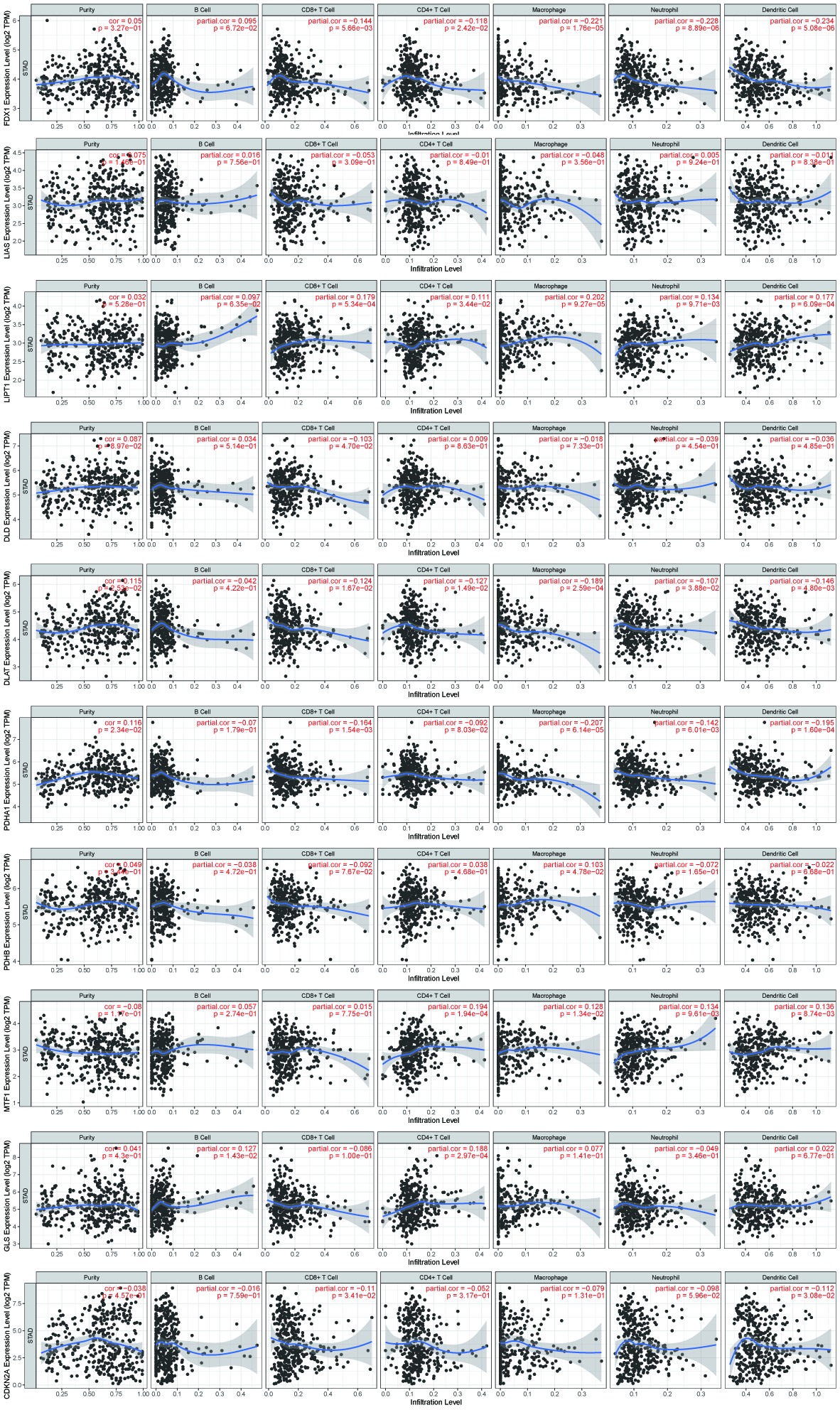

Supplement: Supplementary file 7 — Supplementary file7 [file 10238_2026_2160_MOESM7_ESM.tif]

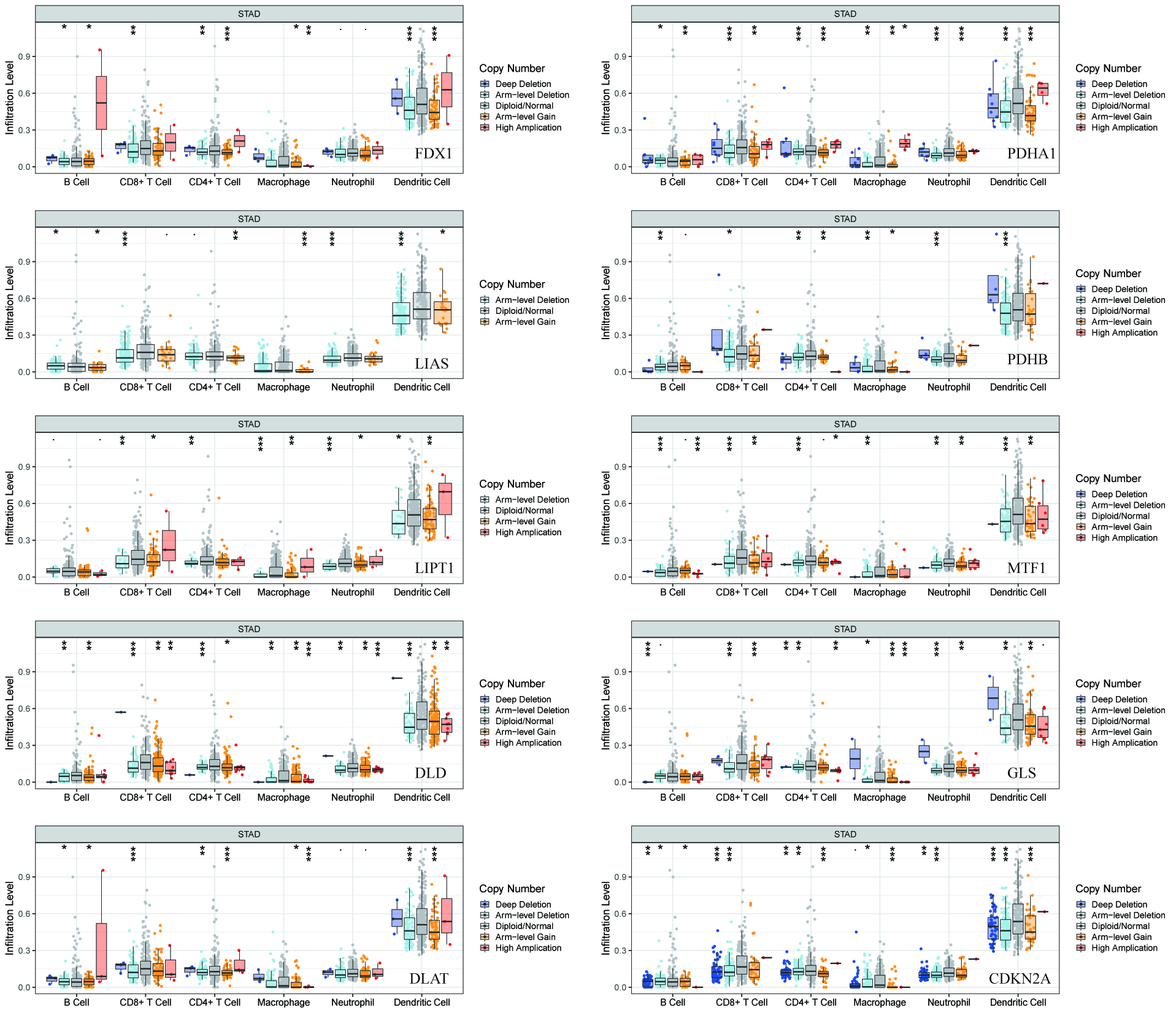

Supplement: Supplementary file 8 — Supplementary file8 [file 10238_2026_2160_MOESM8_ESM.tif]

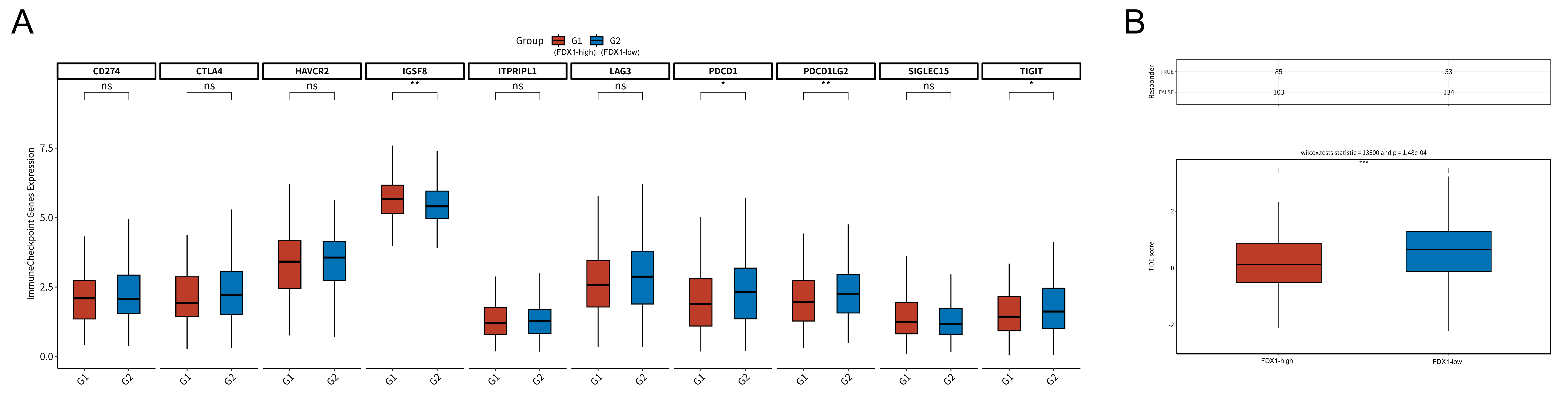

Supplement: Supplementary file 9 — Supplementary file9 [file 10238_2026_2160_MOESM9_ESM.tif]

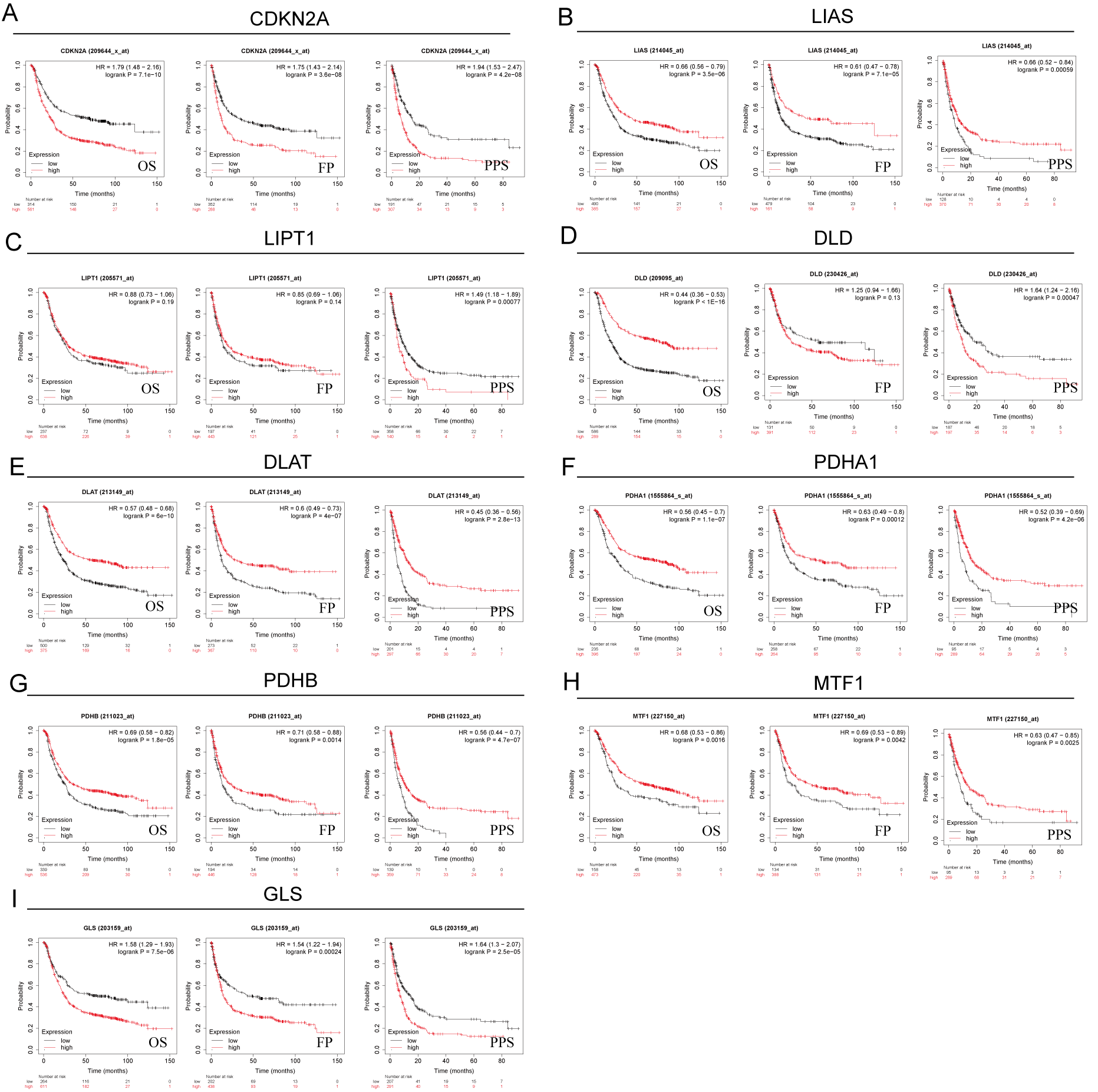

Supplement: Supplementary file 10 — Supplementary file10 [file 10238_2026_2160_MOESM10_ESM.tif]
